# Tracking plasma DNA mutation dynamics in estrogen receptor positive metastatic breast cancer with dPCR-SEQ

**DOI:** 10.1038/s41523-018-0093-3

**Published:** 2018-12-05

**Authors:** Sunil Kumar, Daniel Lindsay, Q. Brent Chen, Amy L. Garrett, Xianming M. Tan, Carey K. Anders, Lisa A. Carey, Gaorav P. Gupta

**Affiliations:** 1 Lineberger Comprehensive Cancer Center, University of North Carolina, Chapel Hill, NC USA; 2 Department of Radiation Oncology, University of North Carolina, Chapel Hill, NC USA; 3 Department of Biostatistics, University of North Carolina, Chapel Hill, NC USA; 4 Division of Hematology/Oncology, University of North Carolina, Chapel Hill, NC USA

## Abstract

Serial monitoring of plasma DNA mutations in estrogen receptor positive metastatic breast cancer (ER + MBC) holds promise as an early predictor of therapeutic response. Here, we developed dPCR-SEQ, a customized assay that utilizes digital PCR-based target enrichment followed by next-generation sequencing to analyze plasma DNA mutations in *ESR1*, *PIK3CA*, and *TP53*. We validated dPCR-SEQ in a prospective cohort of 58 patients with ER + MBC and demonstrate excellent concordance with hotspot *ESR1* mutation abundance measured by conventional digital PCR. The dPCR-SEQ assay revealed *ESR1*, *PIK3CA*, and *TP53* plasma ctDNA mutations in 55%, 32%, and 32% of the study patients, respectively. We also observed dynamic changes in *ESR1*, *PIK3CA*, and *TP53* ctDNA mutant allele fraction (MAF) that were frequently discordant between the different genes. Thus, monitoring plasma DNA mutation dynamics using a dPCR-SEQ assay is feasible, accurate, and may be investigated as a biomarker of therapeutic response in ER + MBC.

## Genomic testing: Blood test reveals mutation dynamics in three cancer-related genes

A new blood test that detects mutations in three cancer-related genes could help guide clinical decision-making for women with aggressive breast tumors. Gaorav Gupta and coworkers from the University of North Carolina at Chapel Hill, USA, developed a droplet-based microfluidic technology that replicates target genomic fragments and then sequences the DNA to quantify the fraction of gene variants that are mutated in the blood sample. The researchers designed the method to look at three genes often mutated in metastatic cases of estrogen receptor–positive breast cancer. They validated the technique using blood samples from 58 patients. In a subset of these cases, they showed that the assay, known as dPCR-SEQ, picked up changes in mutation frequency over time. These mutation dynamics could likely serve as a biomarker of therapeutic response, although clinical validation is needed.

## Introduction

The detection and monitoring of circulating tumor DNA (ctDNA) mutations in ER + MBC patients has emerged as a promising predictive biomarker of therapeutic sensitivity.^1–6^ Digital PCR (dPCR) is an established and cost-effective technology to serially monitor plasma DNA mutation kinetics with exceptional accuracy, sensitivity, and specificity.^7^ However, dPCR is limited to detecting a relatively small number of hotspot target alleles for which primers and/or probes are specifically designed, and is not well suited for the analysis of genes (e.g., *TP53*) where pathogenic mutations are heterogeneous. In contrast, plasma ctDNA NGS-based assays have the potential to identify a broader genomic target region, yet concerns have arisen regarding their sensitivity, specificity, and accuracy of measuring mutant allele fraction (MAF) of target alleles in plasma ctDNA.^8^ Furthermore, the high cost and complexity of bioinformatics analysis of advanced NGS-based assays^9^ are barriers for implementing serial NGS-based assessment of patients over time.

## Results

### Design and implementation of dPCR-SEQ

**Fig. 1:**
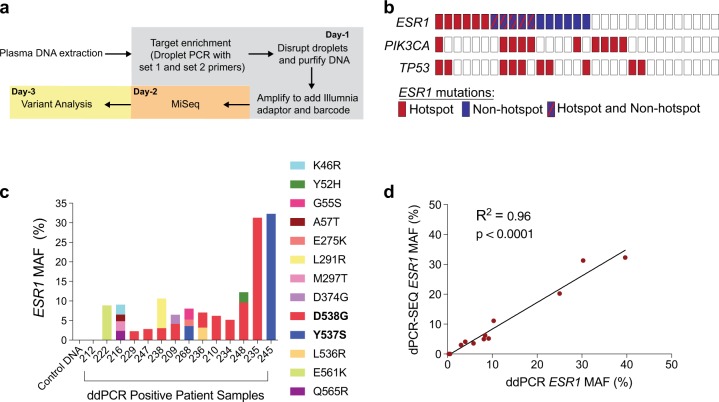
Detection of *ESR1*, *PIK3CA*, and *TP53* mutations in plasma ctDNA from ER + MBC patients. **a** Schematic of the dPCR-SEQ workflow for plasma ctDNA analysis. **b** Summary of plasma ctDNA mutations identified in 31 patients analyzed for the presence of *ESR1*, *PIK3CA*, and *TP53* mutations by dPCR-SEQ. *ESR1* mutations are sub-classified as hotspot or non-hotspot. Some patients had both hotspot and non-hotspot mutations (shown in the shades of red and blue). *PIK3CA* and *TP53* mutations were frequently observed and were consistent with known pathogenic mutations. **c** Mutant allele frequency and identity of *ESR1* coding sequence mutations identified by dPCR-SEQ in patients who were positive by the dPCR *ESR1* hotspot mutation assay. **d** Linear regression analysis of *ESR1* hotspot mutation frequency calculated by dPCR and dPCR-SEQ

Here we report the development of dPCR-SEQ, which utilizes digital PCR technology for target enrichment followed by next-generation sequencing (Fig. 1a, Supplementary Methods). We designed a custom primer set for use in the Raindance Thunderbolts OpenSource platform to perform multiplexed amplification of *ESR1* and *TP53* coding regions and hotspot mutation regions in *PIK3CA*, *PIK3R1*, and *POLE* (Supplementary Table 1). Only mutations in *ESR1*, *PIK3CA*, and *TP53* were recurrently observed in our patient cohort; thus, the analyses presented here are limited to the amplicons targeting these genes in the dPCR-SEQ panel. The amplicons were limited to <120 bp due to the high fragmentation of plasma ctDNA. Up to 16 samples were multiplexed on a MiSeq 2 × 150 bp v3 chip, to achieve mean read depth >3000× (Supplementary Fig. S1a). The FASTQ files were de-multiplexed and aligned to the human reference genome for variant analysis (detailed methods provided in Supplementary Information). The MAF was calculated as the percentage of variant alleles relative to the total sequenced alleles for each position in the target gene coding sequence. A MAF threshold of 1.6% was adequate to eliminate false positive mutations in control genomic DNA when the input material was at least 10 ng (Supplementary Fig. S1b). The streamlined workflow for dPCR-SEQ assay is simple, fast, and cost-effective, with an estimated per sample reagent and sequencing cost of ~$300, and analyzed results available in as few as 3 days.

### Detection of hotspot ESR1 mutations by dPCR

Our goal was to evaluate the feasibility and performance of dPCR-SEQ on a clinical cohort of plasma ctDNA specimens. Towards this goal, we conducted a prospective biomarker study of 58 patients with ER + MBC who had progressed on at least one prior line of endocrine therapy. The prior and current treatment exposures for these patients were heterogeneous, and are described in Supplementary Table S2. In some cases, followup blood samples were obtained while they underwent systemic therapy, typically 4–6 weeks after the first blood sample. The baseline blood samples were analyzed with a multiplexed dPCR assay validated for ultra-sensitive detection of *WT* and hotspot *ESR1* mutations (D538G, Y537S, Y537C, and Y537N, Supplementary Fig. S2a-d). A plasma ctDNA hotspot *ESR1* mutation was identified in 16/58 patients (28%) (Supplementary Fig. 3a). The hotspot *ESR1* MAF was calculated as the percentage of mutant allele relative to the sum of mutant and wild-type alleles detected by dPCR. As expected, the presence of a hotspot *ESR1* mutation in baseline plasma ctDNA correlated with shorter progression-free survival (PFS) (Supplementary Fig. 3b). However, we did not observe a correlation between a reduction in mutation abundance and prolonged PFS (Supplementary Fig. 4a-b) in a limited subset of 11 *ESR1* mutation positive patients with followup blood samples available for analysis. Together with another recent report,^5^ these findings suggest that *ESR1* mutations are frequently subclonal and their dynamics may not reflect the entirety of disease response.

### Comparison of dPCR-SEQ and dPCR in clinical samples

We were able to perform dPCR-SEQ on 31 out of 58 patients (58%) in our cohort due to a recommended assay input of 10 ng plasma DNA. Our study collected 6–8 ml of blood at each timepoint, and it is likely that doubling the volume of blood collected would have substantially increased the proportion of analyzable samples. Mutations in *ESR1*, *PIK3CA*, and/or *TP53* were identified by dPCR-SEQ in 23/31 patients (74%, Fig. 1b). As expected, the most common *ESR1* mutations identified were D538G and Y537S, although we also observed non-hotspot *ESR1* mutations scattered across the *ESR* coding region in 35% of the patient cohort (Supplementary Fig. 5). A similarly high prevalence of non-hotspot *ESR1* mutations were identified in a recent plasma ctDNA NGS study of ER + MBC, whereas they were not identified in ER negative cancer patients.^10^ Further research is necessary to determine if these non-canonical *ESR1* mutations observed in plasma ctDNA are indeed tumor-derived and if they have any impact on therapeutic sensitivity. Consistent with expected results, all of the *PIK3CA* mutations identified were oncogenic hotspot mutations, whereas mutations in the tumor suppressor *TP53* were scattered across the coding sequence and infrequently recurrent (Supplementary Fig. 5).

**Fig. 2:**
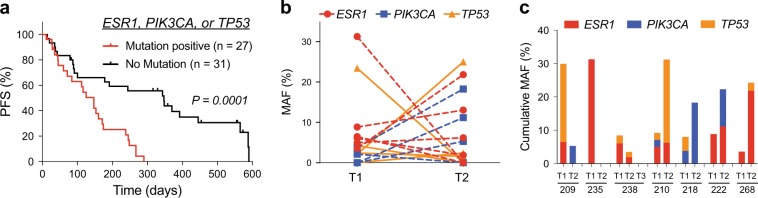
*ESR1*, *PIK3CA* and *TP53* ctDNA mutations in MBC patients. **a** Progression-free survival of patients with any mutation in *ESR1*, *PIK3CA*, or *TP53* relative to patients with none of these mutations detected by either dPCR-SEQ or dPCR. Two-tailed *P* values calculated using the logrank test. **b** Dynamic changes in MAF for *ESR1*, *PIK3CA*, and *TP53* in plasma ctDNA. **c** Stacked histogram plots of cumulative MAF for *ESR1*, *PIK3CA*, and *TP53* in seven patients demonstrate heterogeneous ctDNA mutation dynamics during systemic therapy

Hotspot *ESR1* mutations were identified in 11 out of 31 patients (35%, Fig. 1c), and there was exceptional concordance between the *ESR1* MAF as measured by dPCR-SEQ and by conventional dPCR (Fig. 1d, *R*^2^ = 0.96). Three out of 31 patients had a hotspot *ESR1* mutation detected by dPCR at an abundance that was below the 1.6% detection threshold for dPCR-SEQ. Based on this analysis of hotspot *ESR1* mutations, we estimate that dPCR-SEQ has 79% sensitivity (11/14) and 100% specificity (17/17). Given the increased sensitivity of dPCR relative to dPCR-SEQ, we performed a combined analysis of all 58 patients in our cohort. Patients who had a baseline ctDNA mutation in *ESR1*, *PIK3CA*, and/or *TP53* detected by either assay had significantly worse PFS relative to the remainder of patients in our cohort (Fig. 2a, HR 3.8, 95% CI 1.9–7.6).

### Mutation dynamics in patients undergoing systemic therapy

We were also able to perform dPCR-SEQ at two or more timepoints in seven patients. Matched analyses by dPCR-SEQ in the same patient revealed marked changes in the MAF for *ESR1*, *PIK3CA*, and *TP53* at different timepoints during treatment with systemic therapy (Fig. 2b, c). In three patients (209, 235, and 238), the cumulative MAF decreased at the followup timepoint. Among the four patients who had an increase in cumulative MAF, there were frequently discordant effects among the different mutant alleles, indicating high levels of mutational heterogeneity (Fig. 2c).

## Discussion

Collectively, these findings demonstrate the feasibility and favorable performance profile of a streamlined and cost-effective plasma ctDNA NGS assay that utilizes digital PCR for target enrichment of *ESR1*, *PIK3CA*, and *TP53* coding regions. Our preliminary findings in a cohort of ER + MBC patients suggest that ctDNA mutation dynamics vary significantly between patients and at different timepoints during therapy. Future studies will be necessary to evaluate whether serial monitoring of plasma DNA mutation dynamics using both dPCR and/or NGS may facilitate early detection of therapeutic response in ER + MBC.

## Methods

### Chemicals and reagents

2x TaqMan® Genotyping Master Mix was purchased from Applied Biosystems (Foster City, CA). 125 bp genomic fragments for the *ESR1* wild type locus and the four most common hotspot mutations (p.D538G, p.Y537G, p.Y537S, and p.Y537N) were synthesized as gBlocks from IDT (Supplementary Table S3). These gBlocks were used either directly as positive controls in *ESR1* mutation assay or cloned into a pCR2.1-TOPO TA vector by Topo TA cloning kit (Invitrogen, Carlsbad, CA, USA) according to the manufacturer’s protocol. The gene fragment constructs were verified by Sanger sequencing (Genewiz, New Jersey, USA). Locked Nucleic Acids (LNA)-modified DNA oligonucleotide probes were synthesized by IDT (Integrated DNA Technology, San Jose, CA, see Supplementary Table S3). All LNA probes were 5′ end-labeled with either TET or FAM.

### Patient sample collection and clinical data abstraction

All patients included in this study provided written informed consent to an IRB-approved prospective biomarker study at the University of North Carolina at Chapel Hill Cancer Hospital. The key inclusion criteria were histologically confirmed ER + breast cancer, stage IV disease, and a history of progression on a prior line of endocrine therapy. Patients who developed stage IV disease while on adjuvant endocrine therapy were eligible for this study. De-identified blood samples were collected in the clinical phlebotomy laboratory in coordination with clinician-ordered laboratory tests. Follow-up blood collections were optional and based in part on the following: circulating tumor DNA mutations were identified in the initial blood sample by the lab; coordinator and phlebotomist availability; and patient willingness and ability to provide an additional blood sample. Assay results were not shared with clinical providers. We performed clinical data abstraction using our institutional Electronic Medical Record. PFS was measured from the time of the first blood collection. For the serial timepoint analyses, the first blood sample was collected at the start of a new systemic therapy, and subsequent blood samples were collected at ~4–6 week intervals. Progression of disease was determined by RECIST criteria on cross-sectional imaging or by clinician-based confirmation of symptomatic progression.

### Ethics approval and consent to participate

All patients included in this study provided written informed consent to an IRB-approved prospective biomarker study at the University of North Carolina at Chapel Hill Cancer Hospital.

### Processing of plasma and extraction of circulating DNA

Blood samples were collected in cell-free DNA BCT® blood collection tubes (Streck 218962). Within 1–4 h after blood collection, plasma and cells were separated by two centrifugation steps at 2000 ×*g*. The plasma was stored in 15 ml polypropylene tubes at −80 °C until further use. A QIAamp circulating nucleic acid kit (Qiagen, Valencia, CA, USA; Catalog No. 55114) was used to extract DNA from 2 to 5 ml of plasma according to the manufacturer’s protocol. The amount of purified ctDNA was quantified with a Qubit^®^ fluorometer and PicoGreen quantification reagents (Invitrogen, Carlsbad, CA, USA).

### Analysis of *ESR1* mutations by dPCR

A multiplexed panel was developed to detect hotspot *ESR1* mutations at amino acids 537 and 538. The 5-plex assay panel included a TET-conjugated probe that recognized the wild type allele and four FAM-conjugated probes targeting the p.D538G, p.Y537C, p.Y537N, and p.Y537S alleles (Supplementary Fig S2). The 50 µl pre-PCR assay mixture contained 25 µl of TaqMan® Genotyping Master Mix (Applied Biosystems®) and 2.0 μl Droplet Stabilizer (RainDance^TM^ Technologies, Billerica, MA), along with 0.9 µM of forward (5′-ATCTGTACAGCATGAAGTGCAAGA-3′) and reverse primers (5′-CTAGTGGGCGCATGTAGGC-3′), 0.25 µM of TET-labeled wild-type *ESR1* probe and 0.25 µM of three FAM-labeled probes (p.Y537C, p.Y537N and p.Y537S), 0.50 µM of FAM-labeled p.D538G probe, and 5–50 ng of ctDNA. Emulsified droplets containing the PCR reaction components were generated by the RainDrop® Source chip (RainDance^TM^ Technologies), followed by PCR amplification (Bio-Rad). The PCR cycling parameters were 10 min at 95 °C, then 45 cycles of 95 °C for 15 s and 60 °C for 1 min. A slow ramping speed (0.5 °C) was used during cooling from the denaturation step to the annealing step and the denaturation step immediately following extension. Removing ramping between denaturation to annealing step and using 58 °C extension gave better separation of mutant populations during droplet reading. After PCR amplification, the emulsion was transferred to the RainDrop® Sense instrument (RainDance^TM^ Technologies) to measure the end-point fluorescence signal in each droplet. The recorded fluorescence intensity (“height”) and duration (“width”) through FAM and VIC centered emission filters were analyzed with RainDrop Analyst Software II (RainDance^TM^ Technologies).

### Custom ThunderBolts open source NGS cancer panel

ThunderBolts Open Source System (Raindance^TM^ Technologies, Billerica, MA) (TBOS-NGS-CP) was used for target enrichment and NGS analyses. A custom NGS panel was designed on the ThunderBolts open source option (Raindance) consisting of primers for 136 amplicons with 96 bp mean amplicon size. ThunderBolts open source cancer panel used Raindance’s proprietary algorithm to design mixture of two sets of primers (each set consisted of mixture of 68 forward and 68 reverse primers) for targeted enrichment of *ESR1* (complete CDS), *TP53* (complete CDS), *PIK3CA* (hotspots), *PIK3R1* (hotspots) and *POLE* (exonuclease domain). The complete primer sequences are given in Supplementary Appendix 1. Each primer was synthesized as 25nmol DNA oligo by IDT (Integrated DNA Technology, San Jose, CA). The final pool of primers contained 0.16 nmol of each primer in 10 mM Tris pH 8.0.

### Sample preparation for NGS

The 40 µl reaction mixture consisted of Genotypic master mix (1×), droplet stabilizer (1×), 2 µl of ThunderBolts set 1 or set 2 primers, and 5.0 ng of ctDNA; the mixtures were loaded on each well of a RainDrop® Source chip for droplet generation using the Source instrument (RainDance^TM^ Technologies). The droplet emulsions with the set 1 and set 2 primers for each sample were subject to PCR amplification with the following conditions: 94 °C for 2 min; 55 cycles of 94 °C for 30 s, 54 °C for 30 s, 68 °C for 1 min, and finally one incubation at 68 °C for 10 min. The temperature ramp speed between each step was 1 °C/second. The post-amplification droplets were disrupted by adding 50 µl of droplet destabilizer (RainDance^TM^ Technologies) and vortexing for 30 s followed by centrifugation at 2000× *g* for 2 min. After removal of the oil phase, the amplified DNA was further purified with SPRIselect^TM^ beads in accordance with the ThunderBolts Cancer Panel Manual (RainDance^TM^ Technologies). A limited cycle secondary PCR was performed on the purified DNA to append indexed Illumina adaptors. The 25 µl secondary PCR reaction consisted of 3.25 µl of 10× Platinum Taq Pol HiFi Buffer, 0.875 µl of 50 mM MgSO_4_, 1.124 µl of dNTP (10 mM each), 2.5 µl of 4 M Betaine, 1.25 µl DMSO, 1.25 µl 5 µM Universal forward primer, 1.25 of 5 µM Index reverse primer, 0.5 µl Platinum Taq Pol HiFi, and 13 µl of the first PCR template DNA. The same PCR cycling conditions were used for 10 cycles of amplification. The amplicons were purified with SPRIselect^TM^ beads as per the ThunderBolts Cancer Panel Manual (RainDance^TM^ Technologies). The quantity and quality of the libraries were evaluated using the Bioanalyzer high-sensitivity DNA chip (Agilent Technologies, Palo Alto, CA). The library prepared for each patient sample with distinct barcodes was diluted to 2 nM in nuclease-free water. From this stock, eight samples were pooled in equal volumes. The pooled library was quantified with Qubit® fluorometer and PicoGreen quantification reagents (Invitrogen, Carlsbad, CA, USA) and Bioanalyzer high-sensitivity DNA chip (Agilent Technologies, Palo Alto, CA) before running on MiSeq instrument. DNA libraries prepared from eight samples were pooled for each MiSeq run.

### MiSeq sequencing and data analysis

Each pooled library was sequenced using custom sequencing primers (5′-ACACTCTTTCCCTACACGACGCTCTTCCGATCTCTG-3′ and 5′-GTGACTGGAGTTCAGACGTGTGCTCTTCCGATCTGAC-3′) and the MiSeq Reagent Kit v3 2 × 300 bp on a MiSeq Sequencer (Illumina, San Diego, CA) according to the manufacturer’s instructions. Paired end sequencing with 125 cycles was performed. The FASTQ files were processed to remove adaptors and any low quality bases at the ends of the reads (fastq-mcf, ea-utils-read-only-1.04.636, default parameters except *k* = 2). Whole genome alignments of the adaptor-trimmed reads were performed against the reference genome [hg38] using Bowtie2 (bowtie2–2.2.4, default parameters except --local -N 1 -p 5).

Two groups of aligned reads were then selected. One group had both reads mapped to the same amplicon on the correct strands, had primer sequence matching the amplicon they map to within 1 bp mismatch, and were at least 60 bp in length. The other group had both reads mapped to the correct strand, were at least 60 bp in length, and mapped to different amplicons within 1 kb of each other with primer sequences matching the amplicons to which they mapped within 1 bp mismatch. BAM files were created containing each of these two categories of reads and in which the 5′- and 3′-primer sequences were soft-clipped and the alignment positions were adjusted. The two soft-clipped BAM files were merged and sorted. The resulting single BAM file was converted to mpileup file (samtools-1.19 mpileup with parameters -A -B -d 1000000 -Q 30 -q 20) and subjected to variant calling using VarScan2 (VarScan.v2.3.5.jar mpileup2snp and mpileup2indel, --min-coverage 100 --min-reads2 1 --min-avg-qual 30 --min-var-freq 0 --strand-filter 1 --p-value 0.01 --output-vcf 1). The variants detected by VarScan2 were annotated by snpEff (default parameters). Post-processing of the variants was performed to filter out variants that were also observed in the normal genomic DNA control.

### Statistical analyses

Linear regression and survival analyses were performed in Prism 7 (Graphpad) software. PFS was estimated using the Kaplan–Meier (KM) method. Two-sided log-rank test was applied to compare PFS of different subgroups. Associations between clinical parameters and *ESR1* mutation status were evaluated using a Fisher’s exact test (for categorical variables) and a student’s t-test (for continuous variables).

## Electronic supplementary material

Supplementary Material

## Data Availability

The data generated and analysed during this study are described in the following data record: 10.6084/m9.figshare.c.4299719 (ref ^11^). MiSeq DNA raw sequence files are available in the NCBI Sequence Read Archive (SRA) (SRA accession no. SRP162052). Analysed dPCR results data are available in the figshare repository: 10.6084/m9.figshare.7334507. Additional analysed data files are available in the Supplementary Information of this article

## Electronic supplementary material

**Supplementary information** accompanies the paper on the *npj Breast Cancer* website (10.1038/s41523-018-0093-3).
