# Supplementary material for: Tracking plasma DNA mutation dynamics in estrogen receptor positive metastatic breast cancer with dPCR-SEQ
Source: NPJ Breast Cancer. 2018 Dec 5;4:39. doi: 10.1038/s41523-018-0093-3 (PMC6281679; doi:10.1038/s41523-018-0093-3)

Supplementary Material

Supplementary Table 1: Custom primers used in dPCR-SEQ

Overlap	Customer_Target_ID	RDT_ID	Sense_Primer_ID	Sense_Primer_Name	Sense_Sequence	Sense_Sequence_Illumina_Tail	Antisense_Primer_ID	Antisense_Primer_Name	Antisense_Sequence
-	PIK3CA_01	rdt_68	rdt_68_L	UNC_rdt_68_L	ACCCCCTCCATCAACTTCTT	CGCTCTTCCGATCTCTGACCCCCTCCATCAACTTCTT	rdt_68_R	UNC_rdt_68_R	AGTCGTCTTGTTTCATCAAAAA
-	PIK3CA_02	rdt_69	rdt_69_L	UNC_rdt_69_L	TTTTCAACCCTTTTTAAAAGTAATTG	CGCTCTTCCGATCTCTGTTTTCAACCCTTTTTAAAAGTAATTG	rdt_69_R	UNC_rdt_69_R	GCATTTTAGAATAGGATATTGTATCAT
-	PIK3CA_04	rdt_70	rdt_70_L	UNC_rdt_70_L	AGGTGGAATGAATGGCTGAA	CGCTCTTCCGATCTCTGAGGTGGAATGAATGGCTGAA	rdt_70_R	UNC_rdt_70_R	ACAGAGCAAATGGAAAGGCA
-	PIK3CA_06	rdt_71	rdt_71_L	UNC_rdt_71_L	TCTTTTGATGACATTGCATACA	CGCTCTTCCGATCTCTGTCTTTTGATGACATTGCATACA	rdt_71_R	UNC_rdt_71_R	TGTGCATCATTCATTTGTTTCA
-	POLE_CDS_13	rdt_72	rdt_72_L	UNC_rdt_72_L	GCACACGGCAGCAGG	CGCTCTTCCGATCTCTGGCACACGGCAGCAGG	rdt_72_R	UNC_rdt_72_R	ATGATCCCGTGGAGCTAGAC
-	POLE_CDS_13	rdt_73	rdt_73_L	UNC_rdt_73_L	TCCACGGGATCATAGCCTAG	CGCTCTTCCGATCTCTGTCCACGGGATCATAGCCTAG	rdt_73_R	UNC_rdt_73_R	TGGGTGAAGAGGGACAGTTA
-	POLE_CDS_12	rdt_74	rdt_74_L	UNC_rdt_74_L	ACGGACAGCAGTGAGGAG	CGCTCTTCCGATCTCTGACGGACAGCAGTGAGGAG	rdt_74_R	UNC_rdt_74_R	AGAAGGACAGCCAGGGG
-	POLE_CDS_12	rdt_75	rdt_75_L	UNC_rdt_75_L	TCCTGCTGCATGCTCAGA	CGCTCTTCCGATCTCTGTCCTGCTGCATGCTCAGA	rdt_75_R	UNC_rdt_75_R	ATTAGAGCCTGACCTGCCC
-	POLE_CDS_11	rdt_76	rdt_76_L	UNC_rdt_76_L	AGACACAGACTCACCAGT	CGCTCTTCCGATCTCTGAGACACAGACTCACCAGT	rdt_76_R	UNC_rdt_76_R	GCTCATCTGATCCAAAGGTG
-	POLE_CDS_10	rdt_77	rdt_77_L	UNC_rdt_77_L	TCCCACAATACCGGGTAGTT	CGCTCTTCCGATCTCTGTCCCACAATACCGGGTAGTT	rdt_77_R	UNC_rdt_77_R	CACCCCCAAGCCAGAATATG
-	POLE_CDS_10	rdt_78	rdt_78_L	UNC_rdt_78_L	ACTCAAAATCTTCAATATCTTCTGA	CGCTCTTCCGATCTCTGACTCAAAATCTTCAATATCTTCTGA	rdt_78_R	UNC_rdt_78_R	CTTTGCAGCCTCTGACTTGT
-	POLE_CDS_09	rdt_79	rdt_79_L	UNC_rdt_79_L	TCGATCATGTAGGAAATCATCA	CGCTCTTCCGATCTCTGTCGATCATGTAGGAAATCATCA	rdt_79_R	UNC_rdt_79_R	CCCTGTGGTTTTGGCATTTG
-	POLE_CDS_08	rdt_80	rdt_80_L	UNC_rdt_80_L	ACTGGAAATTTTAGGATGAAGGT	CGCTCTTCCGATCTCTGACTGGAAATTTTAGGATGAAGGT	rdt_80_R	UNC_rdt_80_R	TTTCCGGTAGAAATCACCCG
-	POLE_CDS_08	rdt_81	rdt_81_L	UNC_rdt_81_L	CGGGTGATTTCTACCGGAAA	CGCTCTTCCGATCTCTGCGGGTGATTTCTACCGGAAA	rdt_81_R	UNC_rdt_81_R	TCCTGGATGTAGAACTCTAGT
-	POLE_CDS_07	rdt_82	rdt_82_L	UNC_rdt_82_L	CTCACCACGTGGATCTTCAG	CGCTCTTCCGATCTCTGCTCACCACGTGGATCTTCAG	rdt_82_R	UNC_rdt_82_R	AGAAGATAGCTGACCAGTTG
-	POLE_CDS_07	rdt_83	rdt_83_L	UNC_rdt_83_L	GAGGTTTCCTCTTCATCAGT	CGCTCTTCCGATCTCTGGAGGTTTCCTCTTCATCAGT	rdt_83_R	UNC_rdt_83_R	CCCTGGCAGCTGAGTTAGT
-	POLE_CDS_06	rdt_84	rdt_84_L	UNC_rdt_84_L	CTGTGTACGCGTCGCTG	CGCTCTTCCGATCTCTGCTGTGTACGCGTCGCTG	rdt_84_R	UNC_rdt_84_R	CACTGTGGAGGATCTTGTCAA
-	POLE_CDS_06	rdt_85	rdt_85_L	UNC_rdt_85_L	TGGAAGGACAGCCTGATGTA	CGCTCTTCCGATCTCTGTGGAAGGACAGCCTGATGTA	rdt_85_R	UNC_rdt_85_R	CCTATTTCTCTTGAACCAATGAGC
-	POLE_CDS_05	rdt_86	rdt_86_L	UNC_rdt_86_L	TCACCAAGTCCAGATCCTCTT	CGCTCTTCCGATCTCTGTCACCAAGTCCAGATCCTCTT	rdt_86_R	UNC_rdt_86_R	GTTGTGAGCGAGAAGTTTCAT
-	POLE_CDS_04	rdt_87	rdt_87_L	UNC_rdt_87_L	ATGACACACAGGTCGTCTGA	CGCTCTTCCGATCTCTGATGACACACAGGTCGTCTGA	rdt_87_R	UNC_rdt_87_R	CTCTTAACAGGTGGCTTTGC
-	POLE_CDS_03	rdt_88	rdt_88_L	UNC_rdt_88_L	AAGTGGGTTTTAGCTTGTCG	CGCTCTTCCGATCTCTGAAGTGGGTTTTAGCTTGTCG	rdt_88_R	UNC_rdt_88_R	CTTAGGCAGTGCAGTGGATT
-	POLE_CDS_03	rdt_89	rdt_89_L	UNC_rdt_89_L	CTGCACTGCCTAAGCGC	CGCTCTTCCGATCTCTGCTGCACTGCCTAAGCGC	rdt_89_R	UNC_rdt_89_R	TCTCTAACTGTGTAGAGGATGGT
-	POLE_CDS_02	rdt_90	rdt_90_L	UNC_rdt_90_L	GCATGTTAATGAGCCAGCCT	CGCTCTTCCGATCTCTGGCATGTTAATGAGCCAGCCT	rdt_90_R	UNC_rdt_90_R	GCCTGGAACGGAGTCAGT
-	POLE_CDS_02	rdt_91	rdt_91_L	UNC_rdt_91_L	GTTCCAGGCGCTTGAGTG	CGCTCTTCCGATCTCTGGTTCCAGGCGCTTGAGTG	rdt_91_R	UNC_rdt_91_R	CAGGTGGCATTACAAATTAAGCA
-	POLE_CDS_01	rdt_92	rdt_92_L	UNC_rdt_92_L	CTCCAATTGGCGCCGG	CGCTCTTCCGATCTCTGCTCCAATTGGCGCCGG	rdt_92_R	UNC_rdt_92_R	caaccccacggtgcg
-	ESR1_CDS_01	rdt_93	rdt_93_L	UNC_rdt_93_L	CCCTCCACACCAAAGCATC	CGCTCTTCCGATCTCTGCCCTCCACACCAAAGCATC	rdt_93_R	UNC_rdt_93_R	TCCAGGGGGATCTTGAGC
-	ESR1_CDS_01	rdt_94	rdt_94_L	UNC_rdt_94_L	CCTGGGCGAGGTGTACC	CGCTCTTCCGATCTCTGCCTGGGCGAGGTGTACC	rdt_94_R	UNC_rdt_94_R	GGCCGCGGCGTTGAA
-	ESR1_CDS_01	rdt_95	rdt_95_L	UNC_rdt_95_L	GCAGGTCTACGGTCAGAC	CGCTCTTCCGATCTCTGGCAGGTCTACGGTCAGAC	rdt_95_R	UNC_rdt_95_R	CACGCTGTTGAGTGGGG
-	ESR1_CDS_01	rdt_96	rdt_96_L	UNC_rdt_96_L	CTCCGAGCCCGCTGAT	CGCTCTTCCGATCTCTGCTCCGAGCCCGCTGAT	rdt_96_R	UNC_rdt_96_R	TTCTCCAGGTAGTAGGGCAC
-	ESR1_CDS_01	rdt_97	rdt_97_L	UNC_rdt_97_L	CCAGCGGCTACACGGT	CGCTCTTCCGATCTCTGCCAGCGGCTACACGGT	rdt_97_R	UNC_rdt_97_R	ctccctccTGCCGGG
-	ESR1_CDS_02	rdt_98	rdt_98_L	UNC_rdt_98_L	CCAAATTCAGATAATCGACGCC	CGCTCTTCCGATCTCTGCCAAATTCAGATAATCGACGCC	rdt_98_R	UNC_rdt_98_R	TAGCGAGTCTCCTTGGCAG
-	ESR1_CDS_02	rdt_99	rdt_99_L	UNC_rdt_99_L	GACTCGCTACTGTGCAGTG	CGCTCTTCCGATCTCTGGACTCGCTACTGTGCAGTG	rdt_99_R	UNC_rdt_99_R	ACTTCTCTTGAAGAAGGCCT
-	ESR1_CDS_03	rdt_32	rdt_32_L	UNC_rdt_32_L	TTTTTGTATAAAAGTTTACACGGG	CGCTCTTCCGATCTCTGTTTTTGTATAAAAGTTTACACGGG	rdt_32_R	UNC_rdt_32_R	TCTCCCTCCTCTTCGGTCTTT
-	ESR1_CDS_03	rdt_33	rdt_33_L	UNC_rdt_33_L	GACCGAAGAGGAGGGAGAAT	CGCTCTTCCGATCTCTGGACCGAAGAGGAGGGAGAAT	rdt_33_R	UNC_rdt_33_R	AGCTCTCATGTCTCCAGCA
-	ESR1_CDS_03	rdt_34	rdt_34_L	UNC_rdt_34_L	TGAGAGCTGCCAACCTTTG	CGCTCTTCCGATCTCTGTGAGAGCTGCCAACCTTTG	rdt_34_R	UNC_rdt_34_R	AAGGCACTGACCATCTGG
-	ESR1_CDS_03	rdt_35	rdt_35_L	UNC_rdt_35_L	CTTGTTGGATGCTGAGCCC	CGCTCTTCCGATCTCTGCTTGTTGGATGCTGAGCCC	rdt_35_R	UNC_rdt_35_R	CTGCCAGGTTGGTCAGTAAG
-	ESR1_CDS_03	rdt_36	rdt_36_L	UNC_rdt_36_L	CTGGCAGACAGGGAGCT	CGCTCTTCCGATCTCTGCTGGCAGACAGGGAGCT	rdt_36_R	UNC_rdt_36_R	GTTCTTGAAAAGCTATTGACTCTT
-	ESR1_CDS_04	rdt_37	rdt_37_L	UNC_rdt_37_L	TGAGTCAGCAGGGTTTTTCT	CGCTCTTCCGATCTCTGTGAGTCAGCAGGGTTTTTCT	rdt_37_R	UNC_rdt_37_R	CTAGCCAGGCACATTCTAGA
-	ESR1_CDS_04	rdt_38	rdt_38_L	UNC_rdt_38_L	CCTGGCTAGAGATCCTGATGAT	CGCTCTTCCGATCTCTGCCTGGCTAGAGATCCTGATGAT	rdt_38_R	UNC_rdt_38_R	CTTACCTGTCCAAGAGCAAGT
-	ESR1_CDS_05	rdt_39	rdt_39_L	UNC_rdt_39_L	TGTCTTGTGGAAGattttctgt	CGCTCTTCCGATCTCTGTGTCTTGTGGAAGattttctgt	rdt_39_R	UNC_rdt_39_R	CACCATGCCCTCTACACATT
-	ESR1_CDS_05	rdt_40	rdt_40_L	UNC_rdt_40_L	CGACATGCTGCTGGCTAC	CGCTCTTCCGATCTCTGCGACATGCTGCTGGCTAC	rdt_40_R	UNC_rdt_40_R	CTCACCAGAATTAAGCAAAATAATAGA
-	ESR1_CDS_06	rdt_41	rdt_41_L	UNC_rdt_41_L	TGAGCTtctctctctcactct	CGCTCTTCCGATCTCTGTGAGCTtctctctctcactct	rdt_41_R	UNC_rdt_41_R	ATGGTCCTTCTCTTCCAGAG
-	ESR1_CDS_06	rdt_42	rdt_42_L	UNC_rdt_42_L	ATATCCACCGAGTCCTGGAC	CGCTCTTCCGATCTCTGATATCCACCGAGTCCTGGAC	rdt_42_R	UNC_rdt_42_R	CTGGTGCTGCTGCTGC
-	ESR1_CDS_06	rdt_43	rdt_43_L	UNC_rdt_43_L	CAGCGGCTGGCCCAG	CGCTCTTCCGATCTCTGCAGCGGCTGGCCCAG	rdt_43_R	UNC_rdt_43_R	GGGCATGTTTTCTTTATGTCTCT
-	ESR1_CDS_07	rdt_44	rdt_44_L	UNC_rdt_44_L	CCTACAGTAACAAAGGCATGG	CGCTCTTCCGATCTCTGCCTACAGTAACAAAGGCATGG	rdt_44_R	UNC_rdt_44_R	AGGCGGTGGGCGTCC
-	ESR1_CDS_07	rdt_45	rdt_45_L	UNC_rdt_45_L	CTACATGCGCCCACTAGC	CGCTCTTCCGATCTCTGCTACATGCGCCCACTAGC	rdt_45_R	UNC_rdt_45_R	GCAAGGAATGCGATGAAG
-	ESR1_CDS_07	rdt_46	rdt_46_L	UNC_rdt_46_L	TGCAAAAGTATTACATCACGGG	CGCTCTTCCGATCTCTGTGCAAAAGTATTACATCACGGG	rdt_46_R	UNC_rdt_46_R	TGCAGCAGGGATTATCTGAA
-	TP53_CDS_01	rdt_115	rdt_115_L	UNC_rdt_115_L	TGTCAGTGGGGAACAAGA	CGCTCTTCCGATCTCTGTGTCAGTGGGGAACAAGA	rdt_115_R	UNC_rdt_115_R	CCAAAAAGGGTCAGTCTACC
-	TP53_CDS_02	rdt_116	rdt_116_L	UNC_rdt_116_L	GAGTAGGGCCAGGAAGGG	CGCTCTTCCGATCTCTGGAGTAGGGCCAGGAAGGG	rdt_116_R	UNC_rdt_116_R	CTTGGAACTCAAGGATGCCC
-	TP53_CDS_02	rdt_117	rdt_117_L	UNC_rdt_117_L	CCAAGGCCTCATTCAGCTC	CGCTCTTCCGATCTCTGCCAAGGCCTCATTCAGCTC	rdt_117_R	UNC_rdt_117_R	aggtactgtGTATATACTTACTTCTCC
-	TP53_CDS_03	rdt_118	rdt_118_L	UNC_rdt_118_L	CCTGAAGGGTGAAATATTCTCCA	CGCTCTTCCGATCTCTGCCTGAAGGGTGAAATATTCTCCA	rdt_118_R	UNC_rdt_118_R	TCTTTCCTAGCACTGCCCAA
-	TP53_CDS_04	rdt_119	rdt_119_L	UNC_rdt_119_L	CATAACTGCACCCTTGGTCT	CGCTCTTCCGATCTCTGCATAACTGCACCCTTGGTCT	rdt_119_R	UNC_rdt_119_R	CAAGAAAGGGGAGCCTCAC
-	TP53_CDS_04	rdt_120	rdt_120_L	UNC_rdt_120_L	TCTTGCGGAGATTCTCTTCC	CGCTCTTCCGATCTCTGTCTTGCGGAGATTCTCTTCC	rdt_120_R	UNC_rdt_120_R	TGAGTAGTGGTAATCTACTGGGA
-	TP53_CDS_05	rdt_121	rdt_121_L	UNC_rdt_121_L	CACAGCAGGCCAGTGTG	CGCTCTTCCGATCTCTGCACAGCAGGCCAGTGTG	rdt_121_R	UNC_rdt_121_R	CATGGGCGGCATGAACC
-	TP53_CDS_05	rdt_122	rdt_122_L	UNC_rdt_122_L	CATGCCGCCCATGCAG	CGCTCTTCCGATCTCTGCATGCCGCCCATGCAG	rdt_122_R	UNC_rdt_122_R	GGCCTCATCTTGGGCCT
-	TP53_CDS_06	rdt_123	rdt_123_L	UNC_rdt_123_L	AGGCGGCTCATAGGGC	CGCTCTTCCGATCTCTGAGGCGGCTCATAGGGC	rdt_123_R	UNC_rdt_123_R	CCTCAGCATCTTATCCGAGTG
-	TP53_CDS_07	rdt_124	rdt_124_L	UNC_rdt_124_L	GGGACCCTGGGCAAC	CGCTCTTCCGATCTCTGGGGACCCTGGGCAAC	rdt_124_R	UNC_rdt_124_R	GGAGGTTGTGAGGCGCT
-	TP53_CDS_07	rdt_125	rdt_125_L	UNC_rdt_125_L	CTCACAACCTCCGTCATGTG	CGCTCTTCCGATCTCTGCTCACAACCTCCGTCATGTG	rdt_125_R	UNC_rdt_125_R	GCCCTGTGCAGCTGTG
-	TP53_CDS_07	rdt_126	rdt_126_L	UNC_rdt_126_L	CAGGTCTTGGCCAGTTGG	CGCTCTTCCGATCTCTGCAGGTCTTGGCCAGTTGG	rdt_126_R	UNC_rdt_126_R	TGTGCCCTGACTTTCAACTC
-	TP53_CDS_08	rdt_127	rdt_127_L	UNC_rdt_127_L	GACTTGGCTGTCCCAGAATG	CGCTCTTCCGATCTCTGGACTTGGCTGTCCCAGAATG	rdt_127_R	UNC_rdt_127_R	CCCCTCCTGGCCCCT
-	TP53_CDS_08	rdt_128	rdt_128_L	UNC_rdt_128_L	CGCCGGTGTAGGAGCT	CGCTCTTCCGATCTCTGCGCCGGTGTAGGAGCT	rdt_128_R	UNC_rdt_128_R	GTTCACTGAAGACCCAGGTC
-	TP53_CDS_08	rdt_129	rdt_129_L	UNC_rdt_129_L	ACCATTGTTCAATATCGTCCG	CGCTCTTCCGATCTCTGACCATTGTTCAATATCGTCCG	rdt_129_R	UNC_rdt_129_R	CTGACTGCTCTTTTCACCCA
-	TP53_CDS_09	rdt_130	rdt_130_L	UNC_rdt_130_L	CAGGTCCTcagccccc	CGCTCTTCCGATCTCTGCAGGTCCTcagccccc	rdt_130_R	UNC_rdt_130_R	ACTTTCTGCTCTTGTCTTTCAGA
-	TP53_CDS_10	rdt_131	rdt_131_L	UNC_rdt_131_L	ggggtggtgggCCTG	CGCTCTTCCGATCTCTGggggtggtgggCCTG	rdt_131_R	UNC_rdt_131_R	GCAGTCAGATCCTAGCGTC
-	PIK3R1_01	rdt_64	rdt_64_L	UNC_rdt_64_L	TGTTTTGTGTTTTCATTTCAGGGA	CGCTCTTCCGATCTCTGTGTTTTGTGTTTTCATTTCAGGGA	rdt_64_R	UNC_rdt_64_R	CGCATCTCGTACCAAAAAGG
-	PIK3R1_02	rdt_65	rdt_65_L	UNC_rdt_65_L	TTTACAGGAAAGGGGGAAAT	CGCTCTTCCGATCTCTGTTTACAGGAAAGGGGGAAAT	rdt_65_R	UNC_rdt_65_R	AATTCAACCACAGAACTGAAG
-	PIK3R1_03	rdt_66	rdt_66_L	UNC_rdt_66_L	AAAATTACATGAATATAACACTCAGTT	CGCTCTTCCGATCTCTGAAAATTACATGAATATAACACTCAGTT	rdt_66_R	UNC_rdt_66_R	CCTGGGATGTGCGGGT
-	PIK3R1_04	rdt_67	rdt_67_L	UNC_rdt_67_L	TGACAAACGTATGAACAGCA	CGCTCTTCCGATCTCTGTGACAAACGTATGAACAGCA	rdt_67_R	UNC_rdt_67_R	AGGATTCCATTTCAAATACTTACATCA
-	PIK3CA_01	rdt_0	rdt_0_L	UNC_rdt_0_L	TCGTAAGTGTTACTCAAGAAGCA	CGCTCTTCCGATCTCTGTCGTAAGTGTTACTCAAGAAGCA	rdt_0_R	UNC_rdt_0_R	AAGGGTTGAAAAAGCCGAAG
-	PIK3CA_03	rdt_1	rdt_1_L	UNC_rdt_1_L	GTTATAAATAGTGCACTCAGAATAAAA	CGCTCTTCCGATCTCTGGTTATAAATAGTGCACTCAGAATAAAA	rdt_1_R	UNC_rdt_1_R	AGCATTTGACTTTACCTTATCAA
-	PIK3CA_05	rdt_2	rdt_2_L	UNC_rdt_2_L	GCTCAAAGCAATTTCTACACGA	CGCTCTTCCGATCTCTGGCTCAAAGCAATTTCTACACGA	rdt_2_R	UNC_rdt_2_R	AGCACTTACCTGTGACTCCA

-	PIK3CA_06	rdt_3	rdt_3_L	UNC_rdt_3_L	AGATAAAACTGAGCAAGAGGC	CGCTCTTCCGATCTCTGAGATAAAACTGAGCAAGAGGC	rdt_3_R	UNC_rdt_3_R	TCCAATCCATTTTTGTTGTCCAG
-	POLE_CDS_13	rdt_4	rdt_4_L	UNC_rdt_4_L	CTGCTCCGTGGCCATC	CGCTCTTCCGATCTCTGCTGCTCCGTGGCCATC	rdt_4_R	UNC_rdt_4_R	AGTCATAATCTCAAGGCGGC
-	POLE_CDS_13	rdt_5	rdt_5_L	UNC_rdt_5_L	GCCGCCTTGAGATTATGACT	CGCTCTTCCGATCTCTGGCCGCCTTGAGATTATGACT	rdt_5_R	UNC_rdt_5_R	GCATGTTAGAATCATCCTGGC
-	POLE_CDS_12	rdt_6	rdt_6_L	UNC_rdt_6_L	CATGTGGATGCACTGGGG	CGCTCTTCCGATCTCTGCATGTGGATGCACTGGGG	rdt_6_R	UNC_rdt_6_R	CCGGGCAGCAGTCCA
-	POLE_CDS_11	rdt_7	rdt_7_L	UNC_rdt_7_L	AAATGCTGCCCAGTTACTCA	CGCTCTTCCGATCTCTGAAATGCTGCCCAGTTACTCA	rdt_7_R	UNC_rdt_7_R	CACGTCCAGGAGACCAAAC
-	POLE_CDS_11	rdt_8	rdt_8_L	UNC_rdt_8_L	GTTTGGTCTCCTGGACGTG	CGCTCTTCCGATCTCTGGTTTGGTCTCCTGGACGTG	rdt_8_R	UNC_rdt_8_R	AGCTTGAAGAAGAGAAAGAGCA
-	POLE_CDS_10	rdt_9	rdt_9_L	UNC_rdt_9_L	TGAAGACACAAAAGGGGCC	CGCTCTTCCGATCTCTGTGAAGACACAAAAGGGGCC	rdt_9_R	UNC_rdt_9_R	GCTACCTCATCACCAACAGG
-	POLE_CDS_09	rdt_10	rdt_10_L	UNC_rdt_10_L	ATCCCAGGAGCTTACTTCCC	CGCTCTTCCGATCTCTGATCCCAGGAGCTTACTTCCC	rdt_10_R	UNC_rdt_10_R	TCCTGATGCTGAGACAGACC
-	POLE_CDS_09	rdt_11	rdt_11_L	UNC_rdt_11_L	GGGGCAGTTTGGTCGTC	CGCTCTTCCGATCTCTGGGGGCAGTTTGGTCGTC	rdt_11_R	UNC_rdt_11_R	GGAGGCCTAATGGGGAGTTT
-	POLE_CDS_08	rdt_12	rdt_12_L	UNC_rdt_12_L	GCAAAACTTACAGGTCGTTCA	CGCTCTTCCGATCTCTGGCAAAACTTACAGGTCGTTCA	rdt_12_R	UNC_rdt_12_R	TTTGCAGGCTCATTGGTACA
-	POLE_CDS_07	rdt_13	rdt_13_L	UNC_rdt_13_L	GGAAATTCATGTGAGCAGCG	CGCTCTTCCGATCTCTGGGAAATTCATGTGAGCAGCG	rdt_13_R	UNC_rdt_13_R	GCGAGTACGATGTTCCCTAC
-	POLE_CDS_07	rdt_14	rdt_14_L	UNC_rdt_14_L	GAACATCGTACTCGCGCAT	CGCTCTTCCGATCTCTGGAACATCGTACTCGCGCAT	rdt_14_R	UNC_rdt_14_R	TTCTGCAGAGGGGCGG
-	POLE_CDS_06	rdt_15	rdt_15_L	UNC_rdt_15_L	TTCTGAACCGCTGATGCTTT	CGCTCTTCCGATCTCTGTTCTGAACCGCTGATGCTTT	rdt_15_R	UNC_rdt_15_R	AGAAGAACAGGGAGCAGGAT
-	POLE_CDS_06	rdt_16	rdt_16_L	UNC_rdt_16_L	GTTCTTCTTCACGGCAGGG	CGCTCTTCCGATCTCTGGTTCTTCTTCACGGCAGGG	rdt_16_R	UNC_rdt_16_R	CTTGGTGGGTTTGAAGCGA
-	POLE_CDS_05	rdt_17	rdt_17_L	UNC_rdt_17_L	GCCTCATTTACCCCTGGAAA	CGCTCTTCCGATCTCTGGCCTCATTTACCCCTGGAAA	rdt_17_R	UNC_rdt_17_R	TCCAAGAAGTTTCAGGGCAA
-	POLE_CDS_05	rdt_18	rdt_18_L	UNC_rdt_18_L	CCCTGAAACTTCTTGGAGAGA	CGCTCTTCCGATCTCTGCCCTGAAACTTCTTGGAGAGA	rdt_18_R	UNC_rdt_18_R	GGAAGGAAATGTATTTTACCAATGGA
-	POLE_CDS_04	rdt_19	rdt_19_L	UNC_rdt_19_L	CCTTTCTGGTCGCAATGT	CGCTCTTCCGATCTCTGCCTTTCTGGTCGCAATGT	rdt_19_R	UNC_rdt_19_R	TCTTCTTTCTCATCACCCCT
-	POLE_CDS_03	rdt_20	rdt_20_L	UNC_rdt_20_L	GGGGCTTACCTTAAATCTGCT	CGCTCTTCCGATCTCTGGGGGCTTACCTTAAATCTGCT	rdt_20_R	UNC_rdt_20_R	TCTCTTGTAGACCGAGATTTT
-	POLE_CDS_02	rdt_21	rdt_21_L	UNC_rdt_21_L	GCCATATTCCTGGGTGGG	CGCTCTTCCGATCTCTGGCCATATTCCTGGGTGGG	rdt_21_R	UNC_rdt_21_R	TTTGGTTTTGAGCGGCT
-	POLE_CDS_02	rdt_22	rdt_22_L	UNC_rdt_22_L	TCAAAACCAAACCGCAAATCC	CGCTCTTCCGATCTCTGTCAAAACCAAACCGCAAATCC	rdt_22_R	UNC_rdt_22_R	TTTCACTCAGGGATGATGGC
-	POLE_CDS_01	rdt_23	rdt_23_L	UNC_rdt_23_L	GTCCATCAAGAGGCGCG	CGCTCTTCCGATCTCTGGTCCATCAAGAGGCGCG	rdt_23_R	UNC_rdt_23_R	GTCCCTGCCGGCCAC
-	POLE_CDS_01	rdt_24	rdt_24_L	UNC_rdt_24_L	TGGCCGGCAGGGACC	CGCTCTTCCGATCTCTGTGGCCGGCAGGGACC	rdt_24_R	UNC_rdt_24_R	ACATTTccggccccacc
-	ESR1_CDS_01	rdt_25	rdt_25_L	UNC_rdt_25_L	GGGGACACGGTCTGCA	CGCTCTTCCGATCTCTGGGGGACACGGTCTGCA	rdt_25_R	UNC_rdt_25_R	CTTGGATCTGATGCAGTAGGG
-	ESR1_CDS_01	rdt_26	rdt_26_L	UNC_rdt_26_L	TGGAGCCCCTGAACCG	CGCTCTTCCGATCTCTGTGGAGCCCCTGAACCG	rdt_26_R	UNC_rdt_26_R	TCGGGGTAGTTGTACACGG
-	ESR1_CDS_01	rdt_27	rdt_27_L	UNC_rdt_27_L	TGTACAACTACCCCGAGG	CGCTCTTCCGATCTCTGTGTACAACTACCCCGAGG	rdt_27_R	UNC_rdt_27_R	GACCCGGGGCCGTAG
-	ESR1_CDS_01	rdt_28	rdt_28_L	UNC_rdt_28_L	GTTCGGCTCCAACGGC	CGCTCTTCCGATCTCTGGTTCGGCTCCAACGGC	rdt_28_R	UNC_rdt_28_R	CAGGAAAGGCGACAGCTG
-	ESR1_CDS_01	rdt_29	rdt_29_L	UNC_rdt_29_L	TTTCCTGCAGCCCCACG	CGCTCTTCCGATCTCTGTTTCCTGCAGCCCCACG	rdt_29_R	UNC_rdt_29_R	GTACCTGTAGAATGCCGGC
-	ESR1_CDS_02	rdt_30	rdt_30_L	UNC_rdt_30_L	GTTTTGCTTTTCTAATGTTAATGGA	CGCTCTTCCGATCTCTGGTTTTGCTTTTCTAATGTTAATGGA	rdt_30_R	UNC_rdt_30_R	TGGTACTGGCCAATCTTTCTC
-	ESR1_CDS_02	rdt_31	rdt_31_L	UNC_rdt_31_L	CCAGTACCAATGACAAGGGA	CGCTCTTCCGATCTCTGCCAGTACCAATGACAAGGGA	rdt_31_R	UNC_rdt_31_R	ACTCCATAATGGTAGCCTGAAG
-	ESR1_CDS_02	rdt_100	rdt_100_L	UNC_rdt_100_L	TGGTCCTGTGAGGGCTG	CGCTCTTCCGATCTCTGTGGTCCTGTGAGGGCTG	rdt_100_R	UNC_rdt_100_R	AGGATCTGCTCATAGGATCA
-	ESR1_CDS_03	rdt_101	rdt_101_L	UNC_rdt_101_L	TTTTCCACCTGTGTTTTCAG	CGCTCTTCCGATCTCTGTTTTCCACCTGTGTTTTCAG	rdt_101_R	UNC_rdt_101_R	CCTCCCCATCATCTCTCTGG
-	ESR1_CDS_03	rdt_102	rdt_102_L	UNC_rdt_102_L	CAGAGAGATGATGGGGAGGG	CGCTCTTCCGATCTCTGCAGAGAGATGATGGGGAGGG	rdt_102_R	UNC_rdt_102_R	TCTTAGAGCGTTTGATCATGAG
-	ESR1_CDS_03	rdt_103	rdt_103_L	UNC_rdt_103_L	GAAGAACAGCCTGGCCTTG	CGCTCTTCCGATCTCTGGAAGAACAGCCTGGCCTTG	rdt_103_R	UNC_rdt_103_R	TCTGGTAGGATCATACTCGGA
-	ESR1_CDS_03	rdt_104	rdt_104_L	UNC_rdt_104_L	CCTACCAGACCCTTCAGTGA	CGCTCTTCCGATCTCTGCCTACCAGACCCTTCAGTGA	rdt_104_R	UNC_rdt_104_R	CCTCTTCGCCCAGTTGATC
-	ESR1_CDS_04	rdt_105	rdt_105_L	UNC_rdt_105_L	GCATTAATTTCACCAGTAATGAGTCT	CGCTCTTCCGATCTCTGGCATTAATTTCACCAGTAATGAGTCT	rdt_105_R	UNC_rdt_105_R	CCTGATCATGGAGGGTCAA
-	ESR1_CDS_04	rdt_106	rdt_106_L	UNC_rdt_106_L	TTTGACCCTCCATGATCAGGT	CGCTCTTCCGATCTCTGTTTGACCCTCCATGATCAGGT	rdt_106_R	UNC_rdt_106_R	CTTCCCTGGGTGCTCCAT
-	ESR1_CDS_04	rdt_107	rdt_107_L	UNC_rdt_107_L	GAGCACCCAGGGAAGCTA	CGCTCTTCCGATCTCTGGAGCACCCAGGGAAGCTA	rdt_107_R	UNC_rdt_107_R	TGATCGTAAAGAACATGCTACT
-	ESR1_CDS_05	rdt_108	rdt_108_L	UNC_rdt_108_L	TGTTTTCATAGGAACCAGGG	CGCTCTTCCGATCTCTGTGTTTTCATAGGAACCAGGG	rdt_108_R	UNC_rdt_108_R	TGCAGATTCATCATGCGGAA
-	ESR1_CDS_05	rdt_109	rdt_109_L	UNC_rdt_109_L	TCTGCAGGGAGAGGAGTTTG	CGCTCTTCCGATCTCTGTCTGCAGGGAGAGGAGTTTG	rdt_109_R	UNC_rdt_109_R	AATAAACATTTCATCCAGCATTG
-	ESR1_CDS_06	rdt_110	rdt_110_L	UNC_rdt_110_L	GTGTACACATTTCTGTCCAGC	CGCTCTTCCGATCTCTGGTGTACACATTTCTGTCCAGC	rdt_110_R	UNC_rdt_110_R	TCAGGTGGATCAAAGTGTCTG
-	ESR1_CDS_06	rdt_111	rdt_111_L	UNC_rdt_111_L	ACACTTTGATCCACCTGATG	CGCTCTTCCGATCTCTGACACTTTGATCCACCTGATG	rdt_111_R	UNC_rdt_111_R	ATGTGCCTGATGTGGGAGA
-	ESR1_CDS_07	rdt_112	rdt_112_L	UNC_rdt_112_L	CGGGTTGGCTCTAAAGTAGT	CGCTCTTCCGATCTCTGCGGGTTGGCTCTAAAGTAGT	rdt_112_R	UNC_rdt_112_R	GCACCACGTTCTTGCACT
-	ESR1_CDS_07	rdt_113	rdt_113_L	UNC_rdt_113_L	CTCTATGACCTGCTGCTGGA	CGCTCTTCCGATCTCTGCTCTATGACCTGCTGCTGGA	rdt_113_R	UNC_rdt_113_R	GTGGCTTTGGTCCGTCTC
-	ESR1_CDS_07	rdt_114	rdt_114_L	UNC_rdt_114_L	CTTGGCCACTGCGGG	CGCTCTTCCGATCTCTGCTTGGCCACTGCGGG	rdt_114_R	UNC_rdt_114_R	GCCAGGGAGCTCTCAGAC
-	TP53_CDS_01	rdt_47	rdt_47_L	UNC_rdt_47_L	TCTGTCTTGAACATGAGTTTTT	CGCTCTTCCGATCTCTGTCTGTCTTGAACATGAGTTTTT	rdt_47_R	UNC_rdt_47_R	TGATGTCATCTCTCCTCCCT
-	TP53_CDS_02	rdt_48	rdt_48_L	UNC_rdt_48_L	CCTGGCTCCTTCCCAGC	CGCTCTTCCGATCTCTGCCTGGCTCCTTCCCAGC	rdt_48_R	UNC_rdt_48_R	CTCTGTTGCTGCAGATCCG
-	TP53_CDS_03	rdt_49	rdt_49_L	UNC_rdt_49_L	ACTTTCCACTTGATAAGAGGT	CGCTCTTCCGATCTCTGACTTTCCACTTGATAAGAGGT	rdt_49_R	UNC_rdt_49_R	CCAGCTCCTCTCCCCAG
-	TP53_CDS_03	rdt_50	rdt_50_L	UNC_rdt_50_L	GGGGAGAGGAGCTGGTG	CGCTCTTCCGATCTCTGGGGGAGAGGAGCTGGTG	rdt_50_R	UNC_rdt_50_R	AGACCAAGGGTGCAGTTATG
-	TP53_CDS_04	rdt_51	rdt_51_L	UNC_rdt_51_L	CTTACCTCGCTTAGTGCTCC	CGCTCTTCCGATCTCTGCTTACCTCGCTTAGTGCTCC	rdt_51_R	UNC_rdt_51_R	CCTGTCCTGGGAGAGACC
-	TP53_CDS_04	rdt_52	rdt_52_L	UNC_rdt_52_L	GGCACAAACACGCACCT	CGCTCTTCCGATCTCTGGGCACAAACACGCACCT	rdt_52_R	UNC_rdt_52_R	GGTAGGACCTGATTTCCTTAC
-	TP53_CDS_05	rdt_53	rdt_53_L	UNC_rdt_53_L	CCAGTGTGATGATGGTGAGG	CGCTCTTCCGATCTCTGCCAGTGTGATGATGGTGAGG	rdt_53_R	UNC_rdt_53_R	TGACTGTACCACCATCCACT
-	TP53_CDS_06	rdt_54	rdt_54_L	UNC_rdt_54_L	CTCCCAGAGACCCCAGTT	CGCTCTTCCGATCTCTGCTCCCAGAGACCCCAGTT	rdt_54_R	UNC_rdt_54_R	TGCGTGTGGAGTATTTGGAT
-	TP53_CDS_06	rdt_55	rdt_55_L	UNC_rdt_55_L	CCAAATACTCCACACGCAAATT	CGCTCTTCCGATCTCTGCCAAATACTCCACACGCAAATT	rdt_55_R	UNC_rdt_55_R	GGTCCCCAGGCCTCTG
-	TP53_CDS_07	rdt_56	rdt_56_L	UNC_rdt_56_L	GCTCACCATCGCTATCTGAG	CGCTCTTCCGATCTCTGGCTCACCATCGCTATCTGAG	rdt_56_R	UNC_rdt_56_R	GTCCGCGCCATGGCC
-	TP53_CDS_07	rdt_57	rdt_57_L	UNC_rdt_57_L	GGGCGGGGGTGTGGA	CGCTCTTCCGATCTCTGGGGCGGGGGTGTGGA	rdt_57_R	UNC_rdt_57_R	TTCCTACAGTACTCCCCTGC
-	TP53_CDS_08	rdt_58	rdt_58_L	UNC_rdt_58_L	GGAAGCCAGCCCCTCA	CGCTCTTCCGATCTCTGGGAAGCCAGCCCCTCA	rdt_58_R	UNC_rdt_58_R	AACCTACCAGGGCAGCTAC
-	TP53_CDS_08	rdt_59	rdt_59_L	UNC_rdt_59_L	GGTTTTCTGGGAAGGGACAG	CGCTCTTCCGATCTCTGGGTTTTCTGGGAAGGGACAG	rdt_59_R	UNC_rdt_59_R	GTGGCCCCTGCACCA
-	TP53_CDS_08	rdt_60	rdt_60_L	UNC_rdt_60_L	CTGGCATTCTGGGAGCTTC	CGCTCTTCCGATCTCTGCTGGCATTCTGGGAGCTTC	rdt_60_R	UNC_rdt_60_R	GCAATGGATGATTTGATGCTGT
-	TP53_CDS_08	rdt_61	rdt_61_L	UNC_rdt_61_L	CAAATCATCCATTGCTTGGGA	CGCTCTTCCGATCTCTGCAAATCATCCATTGCTTGGGA	rdt_61_R	UNC_rdt_61_R	gggggctgAGGACCTG
-	TP53_CDS_09	rdt_62	rdt_62_L	UNC_rdt_62_L	TGTCCTTACCAGAACGTTGT	CGCTCTTCCGATCTCTGTGTCCTTACCAGAACGTTGT	rdt_62_R	UNC_rdt_62_R	ACCTGTGGGAAGCGAAAATT
-	TP53_CDS_10	rdt_63	rdt_63_L	UNC_rdt_63_L	GAAAATGTTTCCTGACTCAGAG	CGCTCTTCCGATCTCTGGAAAATGTTTCCTGACTCAGAG	rdt_63_R	UNC_rdt_63_R	TTTCCTCTTGCAGCAGCC
-	PIK3R1_02	rdt_132	rdt_132_L	UNC_rdt_132_L	CCTTATTCCAAAATGTTAATACCTTT	CGCTCTTCCGATCTCTGCCTTATTCCAAAATGTTAATACCTTT	rdt_132_R	UNC_rdt_132_R	CATATTTCCCATCTCGATGAAA
-	PIK3R1_03	rdt_133	rdt_133_L	UNC_rdt_133_L	AATATTGAAGCTGTAGGGAAAAA	CGCTCTTCCGATCTCTGAATATTGAAGCTGTAGGGAAAAA	rdt_133_R	UNC_rdt_133_R	TGTGCGGGTATATTCTTCAT
-	PIK3R1_04	rdt_134	rdt_134_L	UNC_rdt_134_L	AGATTGGAAGAAGACTTGAAGA	CGCTCTTCCGATCTCTGAGATTGGAAGAAGACTTGAAGA	rdt_134_R	UNC_rdt_134_R	TCGTCTTTCTCAGCTGGATAAG
-	PIK3R1_05	rdt_135	rdt_135_L	UNC_rdt_135_L	GCAACCGAAACAAAGCTGAA	CGCTCTTCCGATCTCTGGCAACCGAAACAAAGCTGAA	rdt_135_R	UNC_rdt_135_R	GCATAGCAGCCCTGTTTACT

**Illumin Forward Tail**	CGCTCTTCCGATCTCTG
**Illumin Reverse Tail**	TGCTCTTCCGATCTGAC
**Institution**	UNC_

Antisense_Sequence_Illumina_Tail	Amplicon_Sequence_with_Primers	Plex	Chromosome	Sense_Start	Antisense_Start
TGCTCTTCCGATCTGACAGTCGTCTTGTTTCATCAAAAA	ACCCCCTCCATCAACTTCTTCAAGATGAATCTTCTTACATTTTCGTAAGTGTTACTCAAGAAGCAGAAAGGGAAGAATTTTTTGATGAAACAAGACGACT	1	chr3	179,198,992	179,199,091
TGCTCTTCCGATCTGACGCATTTTAGAATAGGATATTGTATCAT	TTTTCAACCCTTTTTAAAAGTAATTGAACCAGTAGGCAACCGTGAAGAAAAGATCCTCAATCGAGAAATTGGTATGATACAATATCCTATTCTAAAATGC	1	chr3	179,199,107	179,199,206
TGCTCTTCCGATCTGACACAGAGCAAATGGAAAGGCA	AGGTGGAATGAATGGCTGAATTATGATATNTACATTCCTGATCTTCCTCGTGCTGCTCGACTTTGCCTTTCCATTTGCTCTGT	1	chr3	179,209,593	179,209,675
TGCTCTTCCGATCTGACTGTGCATCATTCATTTGTTTCA	TCTTTTGATGACATTGCATACATTCGAAAGACNCTAGCCTTAGATAAAACTGAGCAAGAGGCTTTGGAGTATTTCATGAAACAAATGAATGATGCACA	1	chr3	179,234,200	179,234,297
TGCTCTTCCGATCTGACATGATCCCGTGGAGCTAGAC	GCACACGGCAGCAGGGGCAGCCGGGATGTGGCTTACGTGCCTGGGGCTGCTCCGTGGCCATCCGGCACATGTCCTCNGGGTCTAGCTCCACGGGATCAT	1	chr12	132,673,535	132,673,633
TGCTCTTCCGATCTGACTGGGTGAAGAGGGACAGTTA	TCCACGGGATCATAGCCTAGCTTGGCCTTGGCGGCCGCCTTGAGATTATGACTGCCCACAGGAAGGTAACTGTCCCTCTTCACCCA	1	chr12	132,673,621	132,673,706
TGCTCTTCCGATCTGACAGAAGGACAGCCAGGGG	ACGGACAGCAGTGAGGAGCCATGCTGCTCTGTGGCCCCTACCTGAGGCAGTCCATGTGGATGCACTGGGGCGCCTTGTACTCCCCCTGGCTGTCCTTCT	1	chr12	132,675,357	132,675,455
TGCTCTTCCGATCTGACATTAGAGCCTGACCTGCCC	TCCTGCTGCATGCTCAGACCGTGGACTGCTGCCCGGGCCTCCACAAATGGCCTGGGTTGGAAAGAGGACAGACAAGCAAGTGGGCAGGTCAGGCTCTAAT	1	chr12	132,675,467	132,675,566
TGCTCTTCCGATCTGACGCTCATCTGATCCAAAGGTG	AGACACAGACTCACCAGTCAAAAAAGTCCCCGTTGTAGGTGACCATGATGGTGGGTTTGGTCTCCTGGACGTGTTCAAACCACCTTTGGATCAGATGAGC	1	chr12	132,675,721	132,675,820
TGCTCTTCCGATCTGACCACCCCCAAGCCAGAATATG	TCCCACAATACCGGGTAGTTTCCCAAGTGATACCTCCTTACCTCATCGGGTTCANTGAAGACACAAAAGGGGCCTTCATATTCTGGCTTGGGGGTG	1	chr12	132,676,053	132,676,148
TGCTCTTCCGATCTGACCTTTGCAGCCTCTGACTTGT	ACTCAAAATCTTCAATATCTTCTGAAACAATCTCCCTGTTGGTGATGAGGTAGCCCTAGCNAAGTTCATTAGCAATCAGCACAAGTCAGAGGCTGCAAAG	1	chr12	132,676,150	132,676,249
TGCTCTTCCGATCTGACCCCTGTGGTTTTGGCATTTG	TCGATCATGTAGGAAATCATCATAATCTGGTCTGTCTCAGCATCAGGAAACTTGAGGGGCAGTTTGGTCGTCTCAATGTCAAATGCCAAAACCACAGGG	1	chr12	132,676,553	132,676,651
TGCTCTTCCGATCTGACTTTCCGGTAGAAATCACCCG	ACTGGAAATTTTAGGATGAAGGTAACACAAGCAAAACTTACAGGTCGTTCAACAAGGTCATCTCGGCGGGTGATTTCTACCGGAAA	1	chr12	132,677,322	132,677,407
TGCTCTTCCGATCTGACTCCTGGATGTAGAACTCTAGT	CGGGTGATTTCTACCGGAAAANCATTTCCTCGGTATCTGACATTGTACCAATGAGCCTGCAAAACACACAGTGTGCTAACTAGAGTTCTACATCCAGGA	1	chr12	132,677,388	132,677,486
TGCTCTTCCGATCTGACAGAAGATAGCTGACCAGTTG	CTCACCACGTGGATCTTCAGGTCAATGGAGAGGCGGATGTGGTAGGGAACATCGTACTCGCGCATGTCCACAATGTTGTCCAACTGGTCAGCTATCTTCT	1	chr12	132,677,573	132,677,672
TGCTCTTCCGATCTGACCCCTGGCAGCTGAGTTAGT	GAGGTTTCCTCTTCATCAGTAATGACACCGCCCCTCTGCAGAACACTAGGAATTAACAAGAGAGCAACTAACTCAGCTGCCAGGG	1	chr12	132,677,675	132,677,759
TGCTCTTCCGATCTGACCACTGTGGAGGATCTTGTCAA	CTGTGTACGCGTCGCTGGCGTGATCCTGCTCCCTGTTCTTCTTCACGGCAGGGGAGATCTCCTTCCTCACTTTGACAAGATCCTCCACAGTG	1	chr12	132,679,510	132,679,601
TGCTCTTCCGATCTGACCCTATTTCTCTTGAACCAATGAGC	TGGAAGGACAGCCTGATGTAATTTCGCTTCAAACCCACCAAGTGATTTGGCTATAATGCGAAGAGATCACGCTCATTGGTTCAAGAGAAATAGG	1	chr12	132,679,602	132,679,695
TGCTCTTCCGATCTGACGTTGTGAGCGAGAAGTTTCAT	TCACCAAGTCCAGATCCTCTTTGGGGACAGTCTCCACTTTTGCAATTTTGCCCTGAAACTTCTTGGAGAGAAAAGATGAAACTTCTCGCTCACAAC	1	chr12	132,679,950	132,680,045
TGCTCTTCCGATCTGACCTCTTAACAGGTGGCTTTGC	ATGACACACAGGTCGTCTGACCTGAGTCTATGAAACACACTCACCTTTCTGGTCGCAATGTAGAAATACGGTTTATAGGGCAAAGCCACCTGTTAAGAG	1	chr12	132,680,134	132,680,232
TGCTCTTCCGATCTGACCTTAGGCAGTGCAGTGGATT	AAGTGGGTTTTAGCTTGTCGCAGTCAGGGGCTTACCTTAAATCTGCTTCCGTCATCTTGAATAAAGTAGTAATCCACTGCACTGCCTAAG	1	chr12	132,680,572	132,680,661
TGCTCTTCCGATCTGACTCTCTAACTGTGTAGAGGATGGT	CTGCACTGCCTAAGCGCTTATCTTCATCTAAAATCTCGGTCTACAAGAGAATCAGTCAACACAGACACAAGACCATCCTCTACACAGTTAGAGA	1	chr12	132,680,648	132,680,741
TGCTCTTCCGATCTGACGCCTGGAACGGAGTCAGT	GCATGTTAATGAGCCAGCCTGTCTTCTCACCAGGCTCNTTCAGCCGCTCAAAACCAAACCGCAAATCCATCTTATCCGTCCACTGACTCCGTTCCAGGC	1	chr12	132,681,143	132,681,241
TGCTCTTCCGATCTGACCAGGTGGCATTACAAATTAAGCA	GTTCCAGGCGCTTGAGTGNCGAAACTGAGGAAGTGGCGCCATCATCCCTGAGTGAAAGAAGGGAACCCNGTGCTTAATTTGTAATGCCACCTG	1	chr12	132,681,233	132,681,325
TGCTCTTCCGATCTGACcaaccccacggtgcg	CTCCAATTGGCGCCGGCGCCCGGCGGGGGAGGTGGCCGGCAGGGACCGGNGAAAAGTGTGTGGTAggcgcgcgcggcgcaccgtggggttg	1	chr12	132,687,443	132,687,533
TGCTCTTCCGATCTGACTCCAGGGGGATCTTGAGC	CCCTCCACACCAAAGCATCNGGGATGGCCCTACTGCATCAGATCCAAGGGAACGAGCTGGAGCCCCTGAACCGTCCGCAGCTCAAGATCCCCCTGGA	1	chr6	151,807,923	151,808,019
TGCTCTTCCGATCTGACGGCCGCGGCGTTGAA	CCTGGGCGAGGTGTACCTGGACAGCAGCAAGCCCGCCGTGTACAACTACCCCGAGGGCGCCGCCTACGAGTTCAACGCCGCGGCC	1	chr6	151,808,026	151,808,110
TGCTCTTCCGATCTGACCACGCTGTTGAGTGGGG	GCAGGTCTACGGTCAGACCNGCCTCCCCTACGGCCCCGGGTCTGAGGCTGCNGCGTTCGGCTCCAACGGCCTGGGGGGTTTCCCCCCACTCAACAGCGTG	1	chr6	151,808,122	151,808,221
TGCTCTTCCGATCTGACTTCTCCAGGTAGTAGGGCAC	CTCCGAGCCCGCTGATGCTACTGCACCCGCCGCCGCAGCTGTCGCCTTTCCTGCAGCCCCACGGCCAGCAGGTGCCCTACTACCTGGAGAA	1	chr6	151,808,223	151,808,313
TGCTCTTCCGATCTGACctccctccTGCCGGG	CCAGCGGCTACACGGTGCGCGAGGCCGGCCNGCCGGCATTCTACAGGTACCCGCGCCCGCGCCGCCCGTCGGGGTGGCCGCCGCGCCCGGCAggagggag	1	chr6	151,808,319	151,808,418
TGCTCTTCCGATCTGACTAGCGAGTCTCCTTGGCAG	CCAAATTCAGATAATCGACGCCAGGGTGGCAGAGAAAGATTGGCCAGTACCAATGACAAGGGAAGTATGGCTATGGAATCTGCCAAGGAGACTCGCTA	1	chr6	151,842,598	151,842,695
TGCTCTTCCGATCTGACACTTCTCTTGAAGAAGGCCT	GACTCGCTACTGTGCAGTGTGCAATGACTATGCTTCAGGCTACCATTATGGAGTCTGGTCCTGTGAGGGCTGCAAGGCCTTCTTCAAGAGAAGT	1	chr6	151,842,687	151,842,780
TGCTCTTCCGATCTGACTCTCCCTCCTCTTCGGTCTTT	TTTTTGTATAAAAGTTTACACGGGAAAAAAATAAACTAATTTTTTTTTCCACCTGTGTTTTCAGGGATACGAAAAGACCGAAGAGGAGGGAGA	1	chr6	151,944,109	151,944,201
TGCTCTTCCGATCTGACAGCTCTCATGTCTCCAGCA	GACCGAAGAGGAGGGAGAATGTTGAAACACAAGCGCCAGAGAGATGATGGGGAGGGCAGGGGTGAAGTGGGGTCTGCTGGAGACATGAGAGCT	1	chr6	151,944,184	151,944,276
TGCTCTTCCGATCTGACAAGGCACTGACCATCTGG	TGAGAGCTGCCAACCTTTGGCCAAGCCCGCTCATGATCAAACGCTCTAAGAAGAACAGCCTGGCCTTGTCCCTGACGGCCGACCAGATGGTCAGTGCCTT	1	chr6	151,944,269	151,944,368
TGCTCTTCCGATCTGACCTGCCAGGTTGGTCAGTAAG	CTTGTTGGATGCTGAGCCCCCNATACTCTATTCCGAGTATGATCCTACCAGACCCTTCAGTGAAGCTTCGATGATGGGCTTACTGACCAACCTGGCAG	1	chr6	151,944,366	151,944,463
TGCTCTTCCGATCTGACGTTCTTGAAAAGCTATTGACTCTT	CTGGCAGACAGGGAGCTGGTTCACATGATCAACTGGGCGAAGAGGGTGCCAGGTAAGAATGCGAAGCNCAGCTTTTAAGAGTCAATAGCTTTTCAAGAAC	1	chr6	151,944,457	151,944,556
TGCTCTTCCGATCTGACCTAGCCAGGCACATTCTAGA	TGAGTCAGCAGGGTTTTTCTTGCTTGTTTTCAGGCTTTGTGGATTTGACCCTCCATGATCAGGTCCACCTTCTAGAATGTGCCTGGCTAG	1	chr6	152,011,623	152,011,712
TGCTCTTCCGATCTGACCTTACCTGTCCAAGAGCAAGT	CCTGGCTAGAGATCCTGATGATTGGTCTCGTCTGGCGCTCCATGGAGCACCCAGGGAAGCTACTGTTTGCTCCTAACTTGCTCTTGGACAGGTAAG	1	chr6	152,011,704	152,011,799
TGCTCTTCCGATCTGACCACCATGCCCTCTACACATT	TGTCTTGTGGAAGattttctgtttttttaatctttttatttatttatttatttTTGCTATGTTTTCATAGGAACCAGGGAAAATGTGTAGAGGGCATGGTG	1	chr6	152,060,922	152,061,021
TGCTCTTCCGATCTGACCTCACCAGAATTAAGCAAAATAATAGA	CGACATGCTGCTGGCTACATCATCTCGGTTCCGCATGATGAATCTGCAGGGAGAGGAGTTTGTGTGCCTCAAATCTATTATTTTGCTTAATTCTGGTGAG	1	chr6	152,061,030	152,061,129
TGCTCTTCCGATCTGACATGGTCCTTCTCTTCCAGAG	TGAGCTtctctctctcactctctctctGCGCATTCAGGAGTGTACACATTTCTGTCCAGCACCCTGAAGTCTCTGGAAGAGAAGGACCAT	1	chr6	152,094,348	152,094,437
TGCTCTTCCGATCTGACCTGGTGCTGCTGCTGC	ATATCCACCGAGTCCTGGACAAGATCACAGACACTTTGATCCACCTGATGGCCAAGGCAGGCCTGACCCTGCAGCAGCAGCACCAG	1	chr6	152,094,436	152,094,521
TGCTCTTCCGATCTGACGGGCATGTTTTCTTTATGTCTCT	CAGCGGCTGGCCCAGCTCCTCCTCATCCTCTCCCACATCAGGCACATGAGGTGAGGCATCTGTGGGCTTCCTACAGGAGAGACATAAAGAAAACATGCCC	1	chr6	152,094,519	152,094,618
TGCTCTTCCGATCTGACAGGCGGTGGGCGTCC	CCTACAGTAACAAAGGCATGGAGCATCTGTACAGCATGAAGTGCAAGAACGTGGTGCCCCTCTATGACCTGCTGCTGGAGATGCTGGACGCCCACCGCCT	1	chr6	152,098,725	152,098,824
TGCTCTTCCGATCTGACGCAAGGAATGCGATGAAG	CTACATGCGCCCACTAGCCGTGNAGGGGCNTCCGTGGAGGAGACGGACCAAAGCCACTTGGCCACTGCGGGCTCTACTTCATCGCATTCCTTGC	1	chr6	152,098,823	152,098,916
TGCTCTTCCGATCTGACTGCAGCAGGGATTATCTGAA	TGCAAAAGTATTACATCACGGGGGANGCAGAGGGTTTCCCTGCCACNGTCTGAGAGCTCCCTGGCTCCCACACGGTTCAGATAATCCCTGCTGCA	1	chr6	152,098,914	152,099,008
TGCTCTTCCGATCTGACCCAAAAAGGGTCAGTCTACC	TGTCAGTGGGGAACAAGAAGTGGAGAATGTCAGTCTGAGTCAGGCCCTTCTGTCTTGAACATGAGTTTTTTATGGCGGGNGGTAGACTGACCCTTTTTGG	1	chr17	7,669,580	7,669,679
TGCTCTTCCGATCTGACCTTGGAACTCAAGGATGCCC	GAGTAGGGCCAGGAAGGGGCNGAGGTCACTCACCTGGAGTGAGCCCTGCTCCCCCCTGGCTCCTTCCCAGCCTGGGCATCCTTGAGTTCCAAG	1	chr17	7,670,576	7,670,668
TGCTCTTCCGATCTGACaggtactgtGTATATACTTACTTCTCC	CCAAGGCCTCATTCAGCTCTCGGAACATCTNGAAGCGCTCACGCCCACGGATCTGCAGCAACAGAGGAGGGGGAGAAGTAAGTATATACacagtacct	1	chr17	7,670,664	7,670,761
TGCTCTTCCGATCTGACTCTTTCCTAGCACTGCCCAA	CCTGAAGGGTGAAATATTCTCCATCCAGTGGTTTCTTCTTTGGCTGGGGAGAGGAGCTGGTGTTGTTGGGCAGTGCTAGGAAAGA	1	chr17	7,673,534	7,673,618
TGCTCTTCCGATCTGACCAAGAAAGGGGAGCCTCAC	CATAACTGCACCCTTGGTCTCCTCCACCGCTTCTTGTCCTGCTTGCTTACCTCGCTTAGTGCTCCCTGGGGGCAGCTCGTGGTGAGGCTCCCCTTTCTTG	1	chr17	7,673,651	7,673,750
TGCTCTTCCGATCTGACTGAGTAGTGGTAATCTACTGGGA	TCTTGCGGAGATTCTCTTCCTCTGTGCGCCGGTCTCTCCCAGGACAGGCACAAACACGCACCTCAAAGCTGTTCCGTCCCAGTAGATTACCACTACTCA	1	chr17	7,673,746	7,673,844
TGCTCTTCCGATCTGACCATGGGCGGCATGAACC	CACAGCAGGCCAGTGTGCAGGGTGGCAANTGGCTCCTGACCTGGAGTCTTCCAGTGTGATGATGGTGAGGATGGGCCTCCGGTTCATGCCGCCCATG	1	chr17	7,674,141	7,674,237
TGCTCTTCCGATCTGACGGCCTCATCTTGGGCCT	CATGCCGCCCATGCAGGAACTGTTACACATGTAGTTGTAGTGGATGGTGGTACAGTCAGAGCCAACCTAGGAGATAACACAGGCCCAAGATGAGGCC	1	chr17	7,674,225	7,674,321
TGCTCTTCCGATCTGACCCTCAGCATCTTATCCGAGTG	AGGCGGCTCATAGGGCACCACCACACTATGNCGAAAAGTGTTTCTGTCATCCAAATACTCCACACGCAAATTTCCTTCCACTCGGATAAGATGCTGAGG	1	chr17	7,674,862	7,674,960
TGCTCTTCCGATCTGACGGAGGTTGTGAGGCGCT	GGGACCCTGGGCAACCAGCCCTGTCGTCTCTCCAGCCCNAGCTGCTCACCATCGCTATCTGAGCAGCGCTCATGGTGGGGGCAGCGCCTCACAACCTCC	1	chr17	7,675,004	7,675,102
TGCTCTTCCGATCTGACGCCCTGTGCAGCTGTG	CTCACAACCTCCGTCATGTGCTGTGACTGCTTGTAGATGGCCATGGCGCGGACGCGGGTGCCGGGCGGGGGTGTGGAATCAACCCACAGCTGCACAGGGC	1	chr17	7,675,091	7,675,190
TGCTCTTCCGATCTGACTGTGCCCTGACTTTCAACTC	CAGGTCTTGGCCAGTTGGCAAAACATCTTGTTGAGGGCAGGGGAGTACTGTAGGAAGAGGAAGGAGACAGAGTTGAAAGTCAGGGCACA	1	chr17	7,675,190	7,675,278
TGCTCTTCCGATCTGACCCCCTCCTGGCCCCT	GACTTGGCTGTCCCAGAATGCAAGAAGCCCAGACGGAAACCGTAGCTGCCCTGGTAGGTTTTCTGGGAAGGGACAGAAGATGACAGGGGCCAGGAGGGG	1	chr17	7,676,007	7,676,105
TGCTCTTCCGATCTGACGTTCACTGAAGACCCAGGTC	CGCCGGTGTAGGAGCTGCTGGTGCAGGGGCCACGNGGGGAGCAGCCTCTGGCATTCTGGGAGCTTCATCTGGACCTGGGTCTTCAGTGAAC	1	chr17	7,676,120	7,676,210
TGCTCTTCCGATCTGACCTGACTGCTCTTTTCACCCA	ACCATTGTTCAATATCGTCCGNGGACAGCATCAAATCATCCATTGCTTGGGANGGCAAGGGGGACTGTANATGGGTGAAAAGAGCAGTCAG	1	chr17	7,676,209	7,676,299
TGCTCTTCCGATCTGACACTTTCTGCTCTTGTCTTTCAGA	CAGGTCCTcagccccccagccNNNNNNNNNNNNNNNNNNNNNNNNNNNNNNNNNNNNNNNNNNNNNNNNNNNTGTCCTTACCAGAACGTTGTTTTCAGGAAGTCTGAAAGACAAGAGCAGAAAGT	1	chr17	7,676,305	7,676,425
TGCTCTTCCGATCTGACGCAGTCAGATCCTAGCGTC	ggggtggtgggCCTGCCCTTCCAATGGATCCACTCACAGTTTCCATAGGTCTGAAAATGTTTCCTGACTCAGAGGGGGCTCGACGCTAGGATCTGACTGC	1	chr17	7,676,484	7,676,583
TGCTCTTCCGATCTGACCGCATCTCGTACCAAAAAGG	TGTTTTGTGTTTTCATTTCAGGGAAGAAGTGAATGAAAANACTTCGAGATACAGCAGACGGGACCTTTTTGGTACGAGATGCG	1	chr5	68,293,080	68,293,161
TGCTCTTCCGATCTGACAATTCAACCACAGAACTGAAG	TTTACAGGAAAGGGGGAAATAACAAATTAATCAAAATATTTCATCGAGATGGGAAATATGGCTTNTCTGACCCATTAACCTTCAGTTCTGTGGTTGAATT	1	chr5	68,293,296	68,293,395
TGCTCTTCCGATCTGACCCTGGGATGTGCGGGT	AAAATTACATGAATATAACACTCAGTTTCAAGAAAAAAGTCGAGAATATGATAGATTATATGAAGAATATACCCGCACATCCCAGG	1	chr5	68,293,750	68,293,835
TGCTCTTCCGATCTGACAGGATTCCATTTCAAATACTTACATCA	TGACAAACGTATGAACAGCATTAAACCAGACCTTATCCAGCTGAGAAAGACGAGAGACCAATACTTGATGTAAGTATTTGAAATGGAATCCT	1	chr5	68,295,256	68,295,347
TGCTCTTCCGATCTGACAAGGGTTGAAAAAGCCGAAG	TCGTAAGTGTTACTCAAGAAGCAGAAAGGGAAGAATTTTTTGATGAAACAAGACGACTTTGTGACCTTCGGCTTTTTCAACCCTT	2	chr3	179,199,034	179,199,118
TGCTCTTCCGATCTGACAGCATTTGACTTTACCTTATCAA	GTTATAAATAGTGCACTCAGAATAAAAATTCTTTGTGCAACCTACGTGAATGTAAATATTCGAGACATTGATAAGGTAAAGTCAAATGCT	2	chr3	179,203,715	179,203,804
TGCTCTTCCGATCTGACAGCACTTACCTGTGACTCCA	GCTCAAAGCAATTTCTACACGAGATCCTCTCTCTGAAATCACTGAGCAGGAGAAAGATTTTCTATGGAGTCACAGGTAAGTGCT	2	chr3	179,218,260	179,218,343

TGCTCTTCCGATCTGACTCCAATCCATTTTTGTTGTCCAG	AGATAAAACTGAGCAAGAGGCTTTGGAGTATTTCATGAAACAAATGAATGATGCACATCATGGTGGCTGGACAACAAAAATGGATTGGA	2	chr3	179,234,241	179,234,329
TGCTCTTCCGATCTGACAGTCATAATCTCAAGGCGGC	CTGCTCCGTGGCCATCCGGCACATGTCCTCNGGGTCTAGCTCCACGGGATCATAGCCTAGCTTGGCCTTGGCGGCCGCCTTGAGATTATGACT	2	chr12	132,673,581	132,673,673
TGCTCTTCCGATCTGACGCATGTTAGAATCATCCTGGC	GCCGCCTTGAGATTATGACTGCCCACAGGAAGGTAACTGTCCCTCTTCACCCACCTGGAAGGAGAATGAGAACAGAAGCCAGGATGATTCTAACATGC	2	chr12	132,673,654	132,673,751
TGCTCTTCCGATCTGACCCGGGCAGCAGTCCA	CATGTGGATGCACTGGGGCGCCTTGTACTCCCCCTGGCTGTCCTTCTGGAAGCCTATCTCCTGCTGCATGCTCAGACCGTGGACTGCTGCCCGG	2	chr12	132,675,409	132,675,502
TGCTCTTCCGATCTGACCACGTCCAGGAGACCAAAC	AAATGCTGCCCAGTTACTCATAGAGAAGACACAGACTCACCAGTCAAAAAAGTCCCCGTTGTAGGTGACCATGATGGTGGGTTTGGTCTCCTGGACGTG	2	chr12	132,675,695	132,675,793
TGCTCTTCCGATCTGACAGCTTGAAGAAGAGAAAGAGCA	GTTTGGTCTCCTGGACGTGTTCAAACCACCTTTGGATCAGATGAGCCTGAACCCAAGTCACAGCAGTCAGAGGTCTGCTCTTTCTCTTCTTCAAGCT	2	chr12	132,675,775	132,675,871
TGCTCTTCCGATCTGACGCTACCTCATCACCAACAGG	TGAAGACACAAAAGGGGCCTTCATATTCTGGCTTGGGGGTGAACTCAAAATCTTCAATATCTTCTGAAACAATCTCCCTGTTGGTGATGAGGTAGC	2	chr12	132,676,108	132,676,203
TGCTCTTCCGATCTGACTCCTGATGCTGAGACAGACC	ATCCCAGGAGCTTACTTCCCAGAAGCCACCTGCTCACCTGGCCATCGATCATGTAGGAAATCATCATAATCTGGTCTGTCTCAGCATCAGGA	2	chr12	132,676,509	132,676,600
TGCTCTTCCGATCTGACGGAGGCCTAATGGGGAGTTT	GGGGCAGTTTGGTCGTCTCAATGTCAAATGCCAAAACCACAGGGTCCTGTGGGGACAAAATAAGCATAAAGCCAAGCTCTAAACTCCCCATTAGGCCTCC	2	chr12	132,676,608	132,676,707
TGCTCTTCCGATCTGACTTTGCAGGCTCATTGGTACA	GCAAAACTTACAGGTCGTTCAACAAGGTCATCTCGGCGGGTGATTTCTACCGGAAAANCATTTCCTCGGTATCTGACATTGTACCAATGAGCCTGCAAA	2	chr12	132,677,352	132,677,450
TGCTCTTCCGATCTGACGCGAGTACGATGTTCCCTAC	GGAAATTCATGTGAGCAGCGACCCAACCCTGCCCCACTCACCACGTGGATCTTCAGGTCAATGGAGAGGCGGATGTGGTAGGGAACATCGTACTCGC	2	chr12	132,677,537	132,677,633
TGCTCTTCCGATCTGACTTCTGCAGAGGGGCGG	GAACATCGTACTCGCGCATGTCCACAATGTTGTCCAACTGGTCAGCTATCTTCTTAGAGGTTTCCTCTTCATCAGTAATGACACCGCCCCTCTGCAGAA	2	chr12	132,677,619	132,677,717
TGCTCTTCCGATCTGACAGAAGAACAGGGAGCAGGAT	TTCTGAACCGCTGATGCTTTGCTCACAAGACCAAAGTTTACCTGGAAAGCAGAGCTGTGTACGCGTCGCTGGCGTGATCCTGCTCCCTGTTCTTCT	2	chr12	132,679,456	132,679,551
TGCTCTTCCGATCTGACCTTGGTGGGTTTGAAGCGA	GTTCTTCTTCACGGCAGGGGAGATCTCCTTCCTCACTTTGACAAGATCCTCCACAGTGTGGAAGGACAGCCTGATGTAATTTCGCTTCAAACCCACCAAG	2	chr12	132,679,544	132,679,643
TGCTCTTCCGATCTGACTCCAAGAAGTTTCAGGGCAA	GCCTCATTTACCCCTGGAAAGTCTGGGTGATACTCACCAAGTCCAGATCCTCTTTGGGGACAGTCTCCACTTTTGCAATTTTGCCCTGAAACTTCTTGGA	2	chr12	132,679,917	132,680,016
TGCTCTTCCGATCTGACGGAAGGAAATGTATTTTACCAATGGA	CCCTGAAACTTCTTGGAGAGAAAAGATGAAACTTCTCGCTCACAACCCTAATCAGGATCAGAATGAAAAGGCTTTCCATTGGTAAAATACATTTCCTTCC	2	chr12	132,680,000	132,680,099
TGCTCTTCCGATCTGACTCTTCTTTCTCATCACCCCT	CCTTTCTGGTCGCAATGTAGAAATACGGTTTATAGGGCAAAGCCACCTGTTAAGAGTCACCAACCCATCCAGGGGTGATGAGAAAGAAGA	2	chr12	132,680,177	132,680,266
TGCTCTTCCGATCTGACTCTCTTGTAGACCGAGATTTT	GGGGCTTACCTTAAATCTGCTTCCGTCATCTTGAATAAAGTAGTAATCCACTGCACTGCCTAAGCGCTTATCTTCATCTAAAATCTCGGTCTACAAGAGA	2	chr12	132,680,598	132,680,697
TGCTCTTCCGATCTGACTTTGGTTTTGAGCGGCT	GCCATATTCCTGGGTGGGAGAAGGACCTAGTGCTTACAGGATGCATGTTAATGAGCCAGCCTGTCTTCTCACCAGGCTCNTTCAGCCGCTCAAAACCAAA	2	chr12	132,681,101	132,681,200
TGCTCTTCCGATCTGACTTTCACTCAGGGATGATGGC	TCAAAACCAAACCGCAAATCCATCTTATCCGTCCACTGACTCCGTTCCAGGCGCTTGAGTGNCGAAACTGAGGAAGTGGCGCCATCATCCCTGAGTGAAA	2	chr12	132,681,190	132,681,289
TGCTCTTCCGATCTGACGTCCCTGCCGGCCAC	GTCCATCAAGAGGCGCGCTCCGCCCCTCCCCCGCCCCCCGAGGCGCTCCAATTGGCGCCGGCGCCCGGCGGGGGAGGTGGCCGGCAGGGAC	2	chr12	132,687,398	132,687,488
TGCTCTTCCGATCTGACACATTTccggccccacc	TGGCCGGCAGGGACCGGNGAAAAGTGTGTGGTAggcgcgcgcggcgcaccgtggggttgggaccgggcggcgctgcgggccgcggtggggccggAAATGT	2	chr12	132,687,475	132,687,574
TGCTCTTCCGATCTGACCTTGGATCTGATGCAGTAGGG	GGGGACACGGTCTGCACCCTGCCCGCGGCCACGGACCATGACCATGACCCTCCACACCAAAGCATCNGGGATGGCCCTACTGCATCAGATCCAAG	2	chr6	151,807,876	151,807,970
TGCTCTTCCGATCTGACTCGGGGTAGTTGTACACGG	TGGAGCCCCTGAACCGTCCGCAGCTCAAGATCCCCCTGGAGCGGCCCCTGGGCGAGGTGTACCTGGACAGCAGCAAGCCCGCCGTGTACAACTACCCCGA	2	chr6	151,807,980	151,808,079
TGCTCTTCCGATCTGACGACCCGGGGCCGTAG	TGTACAACTACCCCGAGGGCGCCGCCTACGAGTTCAACGCCGCGGCCGCCGCCAACGCGCAGGTCTACGGTCAGACCNGCCTCCCCTACGGCCCCGGGTC	2	chr6	151,808,064	151,808,163
TGCTCTTCCGATCTGACCAGGAAAGGCGACAGCTG	GTTCGGCTCCAACGGCCTGGGGGGTTTCCCCCACTCAACAGCGTGTCTCCGAGCCCGCTGATGCTACTGCACCCGCCGCCGCAGCTGTCGCCTTTCCTG	2	chr6	151,808,176	151,808,275
TGCTCTTCCGATCTGACGTACCTGTAGAATGCCGGC	TTTCCTGCAGCCCCACGGCCAGCAGGTGCCCTACTACCTGGAGAACGAGCCCAGCGGCTACACGGTGCGCGAGGCCGGCCNGCCGGCATTCTACAGGTAC	2	chr6	151,808,269	151,808,368
TGCTCTTCCGATCTGACTGGTACTGGCCAATCTTTCTC	GTTTTGCTTTTCTAATGTTAATGGATTTACTGTTTTTTTCCCCCAGGCCAAATTCAGATAATCGACGCCAGGGTGGCAGAGAAAGATTGGCCAGTACCA	2	chr6	151,842,550	151,842,649
TGCTCTTCCGATCTGACACTCCATAATGGTAGCCTGAAG	CCAGTACCAATGACAAGGGAAGTATGGCTATGGAATCTGCCAAGGAGACTCGCTACTGTGCAGTGTGCAATGACTATGCTTCAGGCTACCATTATGGAGT	2	chr6	151,842,641	151,842,740
TGCTCTTCCGATCTGACAGGATCTGCTCATAGGATCA	TGGTCCTGTGAGGGCTGCAAGGCCTTCTTCAAGAGAAGTATTCAAGGTAATAGTGTGTTGAAAACGACTTCTATTTTTGATCCTATGAGCAGATCCT	2	chr6	151,842,742	151,842,838
TGCTCTTCCGATCTGACCCTCCCCATCATCTCTCTGG	TTTTCCACCTGTGTTTTCAGGGATACGAAAAGACCGAAGAGGAGGGAGAATGTTGAAACACAAGCGCCAGAGAGATGATGGGGAGG	2	chr6	151,944,153	151,944,238
TGCTCTTCCGATCTGACTCTTAGAGCGTTTGATCATGAG	CAGAGAGATGATGGGGAGGGCAGGGGTGAAGTGGGGTCTGCTGGAGACATGAGAGCTGCCAACCTTTGGCCAAGCCCGCTCATGATCAAACGCTCTAAGA	2	chr6	151,944,220	151,944,319
TGCTCTTCCGATCTGACTCTGGTAGGATCATACTCGGA	GAAGAACAGCCTGGCCTTGTCCCTGACGGCCGACCAGATGGTCAGTGCCTTGTTGGATGCTGAGCCCCCNATACTCTATTCCGAGTATGATCCTACCAGA	2	chr6	151,944,318	151,944,417
TGCTCTTCCGATCTGACCCTCTTCGCCCAGTTGATC	CCTACCAGACCCTTCAGTGAAGCTTCGATGATGGGCTTACTGACCAACCTGGCAGACAGGGAGCTGGTTCACATGATCAACTGGGCGAAGAGG	2	chr6	151,944,409	151,944,501
TGCTCTTCCGATCTGACCCTGATCATGGAGGGTCAA	GCATTAATTTCACCAGTAATGAGTCTTTTTCATNTGAGTCAGCAGGGTTTTTCTTGCTTGTTTTCAGGCTTTGTGGATTTGACCCTCCATGATCAGG	2	chr6	152,011,589	152,011,685
TGCTCTTCCGATCTGACCTTCCCTGGGTGCTCCAT	TTTGACCCTCCATGATCAGGTCCACCTTCTAGAATGTGCCTGGCTAGAGATCCTGATGATTGGTCTCGTCTGGCGCTCCATGGAGCACCCAGGGAAG	2	chr6	152,011,666	152,011,762
TGCTCTTCCGATCTGACTGATCGTAAAGAACATGCTACT	GAGCACCCAGGGAAGCTACTGTTTGCTCCTAACTTGCTCTTGGACAGGTAAGTGACCTGGCTGTAGCTTAGGAGTAGCATGTTCTTTACGATCA	2	chr6	152,011,748	152,011,841
TGCTCTTCCGATCTGACTGCAGATTCATCATGCGGAA	TGTTTTCATAGGAACCAGGGAAAATGTGTAGAGGGCATGGTGGAGATCTTCGACATGCTGCTGGCTACATCATCTCGGTTCCGCATGATGAATCTGCA	2	chr6	152,060,980	152,061,077
TGCTCTTCCGATCTGACAATAAACATTTCATCCAGCATTG	TCTGCAGGGAGAGGAGTTTGTGTGCCTCAAATCTATTATTTTGCTTAATTCTGGTGAGTTGATAACACAAGATAACTCAATGCTGGATGAAATGTTTATT	2	chr6	152,061,072	152,061,171
TGCTCTTCCGATCTGACTCAGGTGGATCAAAGTGTCTG	GTGTACACATTTCTGTCCAGCACCCTGAAGTCTCTGGAAGAGAAGGACCATATCCACCGAGTCCTGGACAAGATCACAGACACTTTGATCCACCTGA	2	chr6	152,094,387	152,094,483
TGCTCTTCCGATCTGACATGTGCCTGATGTGGGAGA	ACACTTTGATCCACCTGATGGCCAAGGCAGGCCTGACCCTGCAGCAGCAGCACCAGCGGCTGGCCCAGCTCCTCCTCATCCTCTCCCACATCAGGCACAT	2	chr6	152,094,466	152,094,565
TGCTCTTCCGATCTGACGCACCACGTTCTTGCACT	CGGGTTGGCTCTAAAGTAGTCCTTTCTGTGNCTTCCCACCTACAGTAACAAAGGCATGGAGCATCTGTACAGCATGAAGTGCAAGAACGTGGTGC	2	chr6	152,098,687	152,098,781
TGCTCTTCCGATCTGACGTGGCTTTGGTCCGTCTC	CTCTATGACCTGCTGCTGGAGATGCTGGACGCCCACCGCCTACATGCGCCCACTAGCCGTGNAGGGGCNTCCGTGGAGGAGACGGACCAAAGCCAC	2	chr6	152,098,784	152,098,879
TGCTCTTCCGATCTGACGCCAGGGAGCTCTCAGAC	CTTGGCCACTGCGGGCTCTACTTCATCGCATTCCTTGCAAAAGTATTACATCACGGGGGANGCAGAGGGTTTCCCTGCCACNGTCTGAGAGCTCCCTGGC	2	chr6	152,098,879	152,098,978
TGCTCTTCCGATCTGACTGATGTCATCTCTCCTCCCT	TCTGTCTTGAACATGAGTTTTTTATGGCGGGNGGTAGACTGACCCTTTTTGGACTTCAGGTGGCTGTAGGAGACAGAAGCAGGGAGGAGAGATGACATCA	2	chr17	7,669,628	7,669,727
TGCTCTTCCGATCTGACCTCTGTTGCTGCAGATCCG	CCTGGCTCCTTCCCAGCCTGGGCATCCTTGAGTTCCAAGGCCTCATTCAGCTCTCGGAACATCTNGAAGCGCTCACGCCCACGGATCTGCAGCAACAGAG	2	chr17	7,670,630	7,670,729
TGCTCTTCCGATCTGACCCAGCTCCTCTCCCCAG	ACTTTCCACTTGATAAGAGGTCCCNAGACTTAGTACCTGAAGGGTGAAATATTCTCCATCCAGTGGTTTCTTCTTTGGCTGGGGAGAGGAGCTGG	2	chr17	7,673,499	7,673,593
TGCTCTTCCGATCTGACAGACCAAGGGTGCAGTTATG	GGGGAGAGGAGCTGGTGTTGTTGGGCAGTGCTAGGAAAGAGGCAAGGAAAGGTGATAAAAGTGAATCTGAGGCATAACTGCACCCTTGGTCT	2	chr17	7,673,579	7,673,670
TGCTCTTCCGATCTGACCCTGTCCTGGGAGAGACC	CTTACCTCGCTTAGTGCTCCCTGGGGGCAGCTCGTGGTGAGGCTCCCCTTTCTTGCGGAGATTCTCTTCCTCTGTGCGCCGGTCTCTCCCAGGACAGG	2	chr17	7,673,696	7,673,793
TGCTCTTCCGATCTGACGGTAGGACCTGATTTCCTTAC	GGCACAAACACGCACCTCAAAGCTGTTCCGTCCCAGTAGATTACCACTACTCAGGATAGGAAAAGAGAAGCAAGAGGCNGTAAGGAAATCAGGTCCTACC	2	chr17	7,673,792	7,673,891
TGCTCTTCCGATCTGACTGACTGTACCACCATCCACT	CCAGTGTGATGATGGTGAGGATGGGCCTCCGGTTCATGCCGCCCATGCAGGAACTGTTACACATGTAGTTGTAGTGGATGGTGGTACAGTCA	2	chr17	7,674,191	7,674,282
TGCTCTTCCGATCTGACTGCGTGTGGAGTATTTGGAT	CTCCCAGAGACCCCAGTTGCAAACCAGACCTCAGGCGGCTCATAGGGCACCACCACACTATGNCGAAAAGTGTTTCTGTCATCCAAATACTCCACACGCA	2	chr17	7,674,830	7,674,929
TGCTCTTCCGATCTGACGGTCCCCAGGCCTCTG	CCAAATACTCCACACGCAAATTTCCTTCCACTCGGATAAGATGCTGAGGAGGGGCCAGACCTAAGAGCAATCNGTGAGGAATCAGAGGCCTGGGGACC	2	chr17	7,674,912	7,675,009
TGCTCTTCCGATCTGACGTCCGCGCCATGGCC	GCTCACCATCGCTATCTGAGCAGCGCTCATGGTGGGGGCAGCGCCTCACAACCTCCGTCATGTGCTGTGACTGCTTGTAGATGGCCATGGCGCGGAC	2	chr17	7,675,047	7,675,143
TGCTCTTCCGATCTGACTTCCTACAGTACTCCCCTGC	GGGCGGGGGTGTGGAATCAACCCACAGCTGCACAGGGCAGGTCTTGGCCAGTTGGCAAAACATCTTGTTGAGGGCAGGGGAGTACTGTAGGAA	2	chr17	7,675,153	7,675,245
TGCTCTTCCGATCTGACAACCTACCAGGGCAGCTAC	GGAAGCCAGCCCCTCAGGGCAACTGACCGTGCAAGTCACAGACTTGGCTGTCCCAGAATGCAAGAAGCCCAGACGGAAACCGTAGCTGCCCTGGTAGGTT	2	chr17	7,675,967	7,676,066
TGCTCTTCCGATCTGACGTGGCCCCTGCACCA	GGTTTTCTGGGAAGGGACAGAAGATGACAGGGGCCAGGAGGGGGCTGGTGCAGGGGCCGCCGGTGTAGGAGCTGCTGGTGCAGGGGCCAC	2	chr17	7,676,063	7,676,152
TGCTCTTCCGATCTGACGCAATGGATGATTTGATGCTGT	CTGGCATTCTGGGAGCTTCATCTGGACCTGGGTCTTCAGTGAACCATTGTTCAATATCGTCCGNGGACAGCATCAAATCATCCATTGC	2	chr17	7,676,167	7,676,254
TGCTCTTCCGATCTGACgggggctgAGGACCTG	CAAATCATCCATTGCTTGGGANGGCAAGGGGGACTGTANATGGGTGAAAAGAGCAGTCAGANGACCAGGTCCTcagccccc	2	chr17	7,676,240	7,676,320
TGCTCTTCCGATCTGACACCTGTGGGAAGCGAAAATT	TGTCCTTACCAGAACGTTGTTTTCAGGAAGTCTGAAAGACAAGAGCAGAAAGTCAGTCCCATGNAATTTTCGCTTCCCACAGGT	2	chr17	7,676,373	7,676,456
TGCTCTTCCGATCTGACTTTCCTCTTGCAGCAGCC	GAAAATGTTTCCTGACTCAGAGGGGGCTCGACGCTAGGATCTGACTGCGGCTCCTCCATGGCAGTGACCCGGAAGGCAGTCTGGCTGCTGCAAGAGGAAA	2	chr17	7,676,536	7,676,635
TGCTCTTCCGATCTGACCATATTTCCCATCTCGATGAAA	CCTTATTCCAAAATGTTAATACCTTTATTTTTATATTGTTTTTACAGGAAAGGGGGAAATAACAAATTAATCAAAATATTTCATCGAGATGGGAAATATG	2	chr5	68,293,256	68,293,355
TGCTCTTCCGATCTGACTGTGCGGGTATATTCTTCAT	AATATTGAAGCTGTAGGGAAAAAATTACATGAATATAACACTCAGTTTCAAGAAAAAAGTCGAGAATATGATAGATTATATGAAGAATATACCCGCACA	2	chr5	68,293,730	68,293,828
TGCTCTTCCGATCTGACTCGTCTTTCTCAGCTGGATAAG	AGATTGGAAGAAGACTTGAAGAAGCAGGCAGCTGAGTATCGAGAAATTGACAAACGTATGAACAGCATTAAACCAGACCTTATCCAGCTGAGAAAGACGA	2	chr5	68,295,209	68,295,308
TGCTCTTCCGATCTGACGCATAGCAGCCCTGTTTACT	GCAACCGAAACAAAGCTGAAAACCTGTTGCGAGGGAAGCGAGATGGCACTTTTCTTGTCCGGGAGAGCAGTAAACAGGGCTGCTATGC	2	chr5	68,296,242	68,296,329

Supplementary Table S2: Patient characteristics. None of the differences between groups are statistically significant ($p<0.05$) using a two-tailed Fisher's exact test (categorical parameters) or a student's t-test (continuous parameters).

**Baseline characteristics**	**ESR1 Hotspot – (n=42)**	**ESR1 Hotspot + (n=16)**
Age	62 (39-90)	54 (38-75)
Ethnicity		
Hispanic	1 (2%)	0
Non-Hispanic White	37 (88%)	14 (88%)
Non-white	4 (10%)	2 (12%)
Smoking History		
Yes	17 (40%)	10 (63%)
No	25 (60%)	6 (37%)
Prior systemic therapy exposure		
Adjuvant endocrine therapy	42	16
Adjuvant tamoxifen	27	10
Adjuvant non-steroidal AI	14	6
Adjuvant exemestane	1	0
>1 line of endocrine therapy for stage IV disease	33 (79%)	13 (81%)
Prior chemotherapy	31 (74%)	14 (88%)
Systemic therapy at study enrollment		
Tamoxifen/non-steroidal AI	9 (21%)	4 (25%)
Exemestane	2 5%)	2 (13%)
Exemestane + everolimus	4 (10%)	3 (19%)
Fulvestrant	7 (17%)	2 (13%)
Letrozole + Cdk4/6 inhibitor	9 (21%)	1 (6%)
Fulvestrant + Cdk4/6 inhibitor	5 (12%)	2 (13%)
Chemotherapy/other	6 (14%)	2 (13%)
Location of metastatic disease		
Bone only	5 (12%)	4 (25%)
Non-bone only	6 (14%)	3 (19%)
Bone and non-bone	31 (74%)	9 (56%)
Histology		
IDC	33 (79%)	13 (81%)
ILC	8 (19%)	1 (6%)
Unknown	1 (2%)	2 (13%)
Receptor status		
ER/PR+ HER2-	33 (79%)	9 (56%)
ER/PR+ HER2+	4 (10%)	4 (25%)
HER2 unknown	5 (12%)	3 (19%)
Median time from diagnosis to metastasis (range)	5.1 (0-21.4) years	3.3 (0-27.8) years
Median time from metastasis to enrollment (range)	1.5 (0.1-9.7) years	3.4 (0.4-8.7) years
Median time from diagnosis to enrollment (range)	7.1 (0.1-21.8) years	9.3 (2.4-36.2) years

Supplementary Table S3: *ESR1* gBlocks and LNA probes

**gBlocks**	**Sequence (**5'-gene-specific sequence-3')	**bp**
ESR1-WT-gBlock	TAACAAAGGCATGGAGCATCTGTACAGCATGAAG TGCAAGAACGTGGTGCCCCTCTATGACCTGCTGCT GGAGATGCTGGACGCCCACCGCCTACATGCGCCC ACTAGCCGTGGAGGGGCATCCG	125
ESR1-Y537C-gBlock	TAACAAAGGCATGGAGCATCTGTACAGCATGAAG TGCAAGAACGTGGTGCCCCTCTGTGACCTGCTGCT GGAGATGCTGGACGCCCACCGCCTACATGCGCCC ACTAGCCGTGGAGGGGCATCCG	125
ESR1-Y537N-gBlock	TAACAAAGGCATGGAGCATCTGTACAGCATGAAG TGCAAGAACGTGGTGCCCCTCAATGACCTGCTGCT GGAGATGCTGGACGCCCACCGCCTACATGCGCCC ACTAGCCGTGGAGGGGCATCCG	125
ESR1-Y537S-gBlock	TAACAAAGGCATGGAGCATCTGTACAGCATGAAG TGCAAGAACGTGGTGCCCCTCTCTGACCTGCTGCT GGAGATGCTGGACGCCCACCGCCTACATGCGCCC ACTAGCCGTGGAGGGGCATCCG	125
ESR1-D538G-gBlock	TAACAAAGGCATGGAGCATCTGTACAGCATGAAG TGCAAGAACGTGGTGCCCCTCTATGGCCTGCTGCT GGAGATGCTGGACGCCCACCGCCTACATGCGCCC ACTAGCCGTGGAGGGGCATCCG	125

**Probe name**	Probe sequence	Tm (°C)a
*ESR1*_TET_WT	5'-/5TET/T+C+T+AT+G+A+CCTG/3IABkFQ/-3'	52.4
*ESR1*_ FAM_Y537C	/56-FAM/CC+T+CT+G+T+GA+C/3IABkFQ/	52.9
*ESR1*_ FAM_Y537N	/56-FAM/C+CCT+C+A+ATG+ACC/3IABkFQ/	54
*ESR1*_ FAM_Y537S	/56-FAM/CCC+TC+T+C+TG+ACCT/3IABkFQ/	58.4
*ESR1*_FAM_D538G	/56-FAM/CTA+T+G+GCC+TGC/3IABkFQ/	56

[a]The melting temperatures (T_m) were predicted using DNA Thermodynamics and Hybridization tool at IDT's website.

Supplementary Fig. S1: Sensitivity and limit of detection of dPCR-SEQ. (a) dPCR-SEQ was designed to amplify target regions with amplicon size <120bp. The multiplexed samples were sequenced by MiSeq. The observed mean read depth for 10 ng sheared genomic DNA was 3800 X. (b) Human genomic DNA from multiple anonymous donors (Promega, Madison, WI; Catalog No. G1521) was used as a reference sample to define the limit of detection. Promega control DNA was used at 1 ng, 2.5 ng, 5 ng, 7.5 ng and 10 ng. We have used 10 ng of ctDNA in dPCR-SEQ. Using a threshold of 1.6% MAF, we did not observe any false-positive mutations when analyzing control DNA with 10 ng input. With lower input quantities, we observed a higher background rate of mutations above the 1.6% MAF threshold.

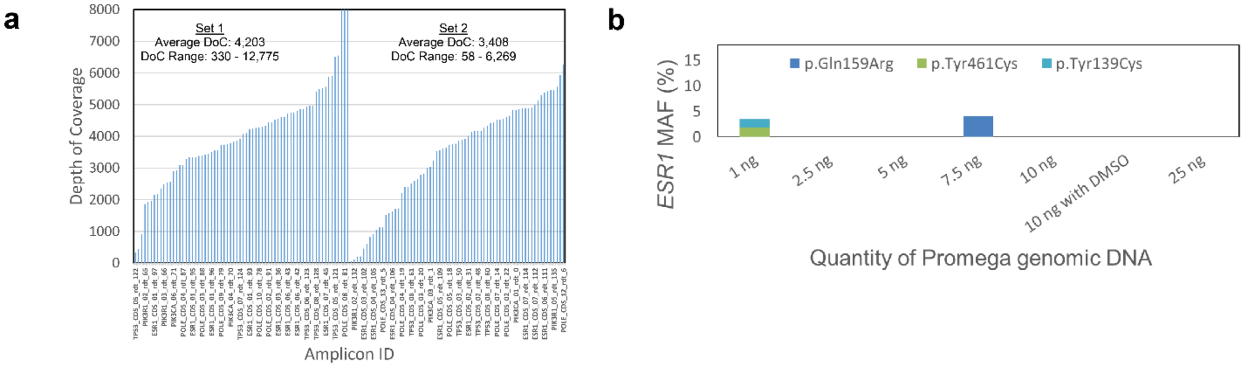

Supplementary Fig. S2: Multiplexed dPCR based *ESR1* assay. (a) A schematic for the multiplexed dPCR reaction. Highly specific FAM or TET conjugated Locked Nucleic Acid probes were developed to detect four hotspot *ESR1* mutations and the wild type (WT) allele. AF-1, activation function-1; DBD, DNA-binding domain; NLS, nuclear localization signal; LBD, ligand-binding domain; AF-2, activation function-2. (b) Titration of *ESR1*-D538G mutants was performed by mixing varying amount of mutants and keeping the WT copies constant. A plasmid containing a 125 bp fragment of the *ESR1* coding sequence containing the D538G mutation was used in this experiment. (c) Highly sensitive detection of D538G copies by dPCR in a titration analysis by spiking sheared genomic DNA from MCF-7 cells with varying amounts of mutant *ESR1*. (d) Two-dimensional dotplot of the 5-plex dPCR assay to detect D538G, Y537C, Y537N, and Y537S mutations and *WT ESR1* using the Raindrop Sense machine (Raindance Technologies).

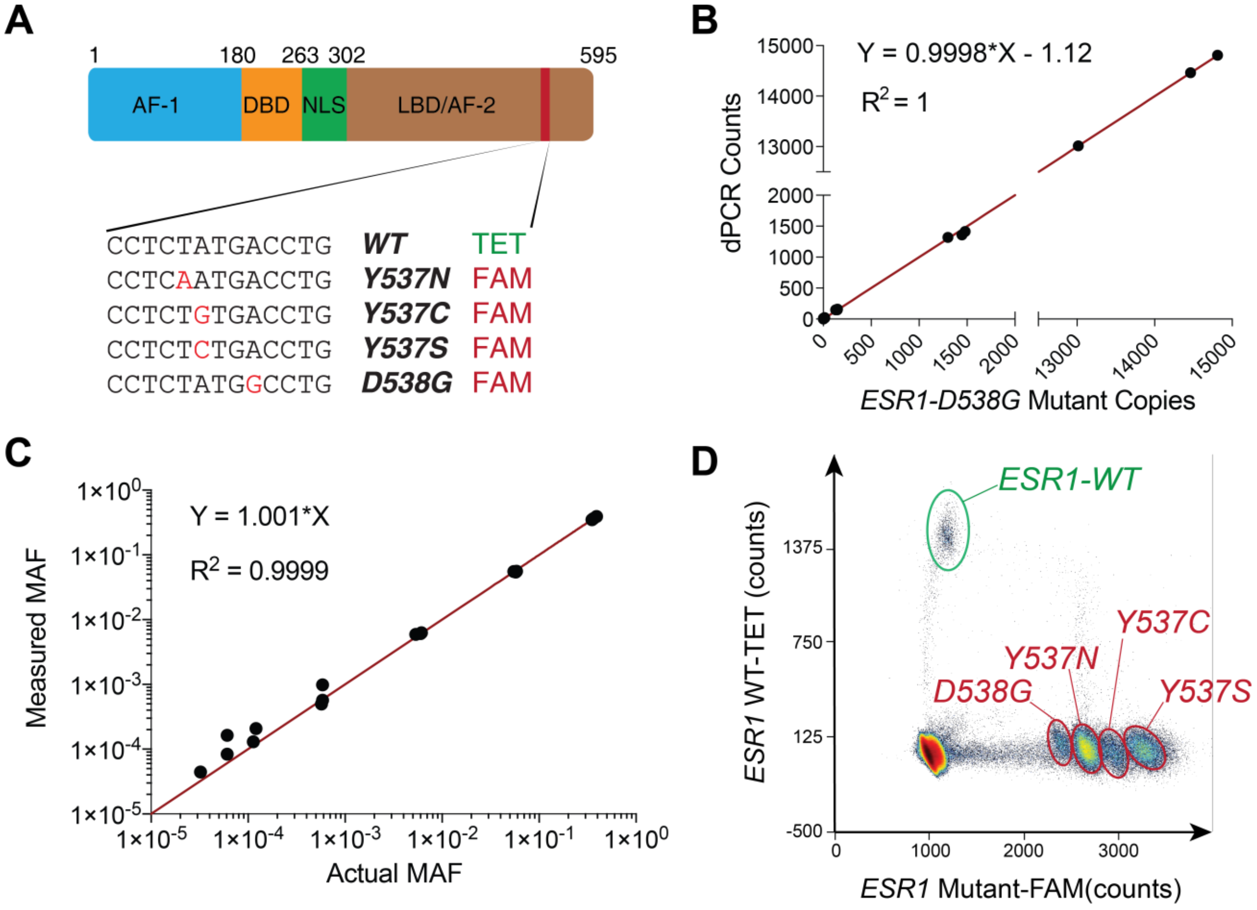

Supplementary Fig. S3: Detection mutations in plasma ctDNA from ER+ MBC patients. (a) *ESR1* mutant allele frequency (%) in 58 patients. (b) Kaplan-Meier progression-free survival plot for MBC patients stratified by detection of plasma *ESR1* hotspot mutations in the baseline blood sample collected for patients in the study. Two-tailed p value measured using the logrank test.

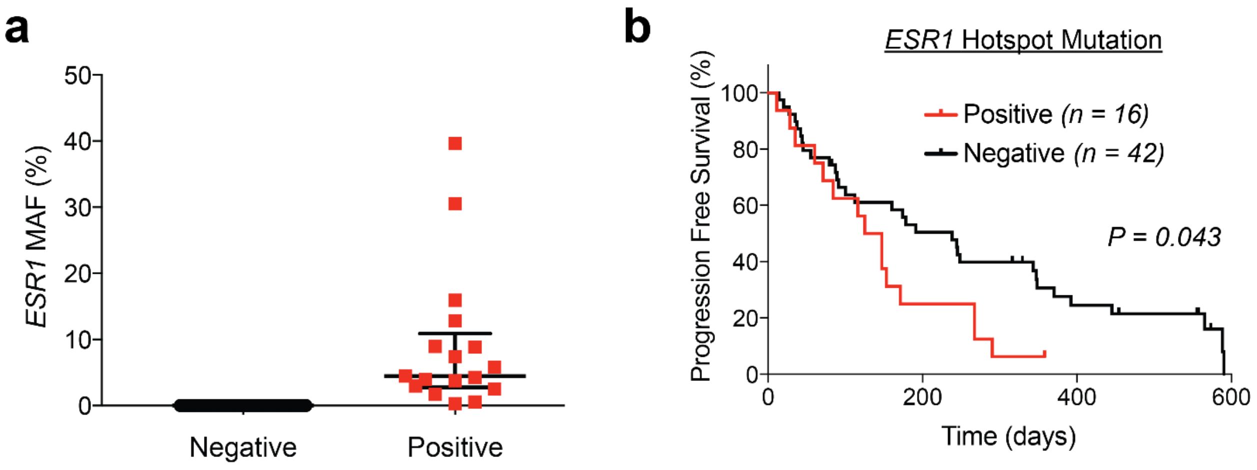

Supplementary Fig. S4: Hotspot *ESR1* mutant allele frequency in serial plasma samples from MBC patients. (a) Dynamic changes in *ESR1* mutant allele frequency in follow-up plasma ctDNA samples from *ESR1* mutation positive patients. Out of 11 patients with longitudinal blood samples available, 6 patients had an increase in the *ESR1* MAF (i.e., "Non-responders"), 4 patients had a reduction in the *ESR1* MAF (i.e., "Responders"), and one patient had unchanged *ESR1* MAF (i.e., "Indeterminate") (b) Progression-free survival in "responder" and "non-responder" patients, stratified by changes in *ESR1* MAF in ctDNA. Survival time is calculated from collection of the first blood sample. Two-tailed p value measured using the logrank test.

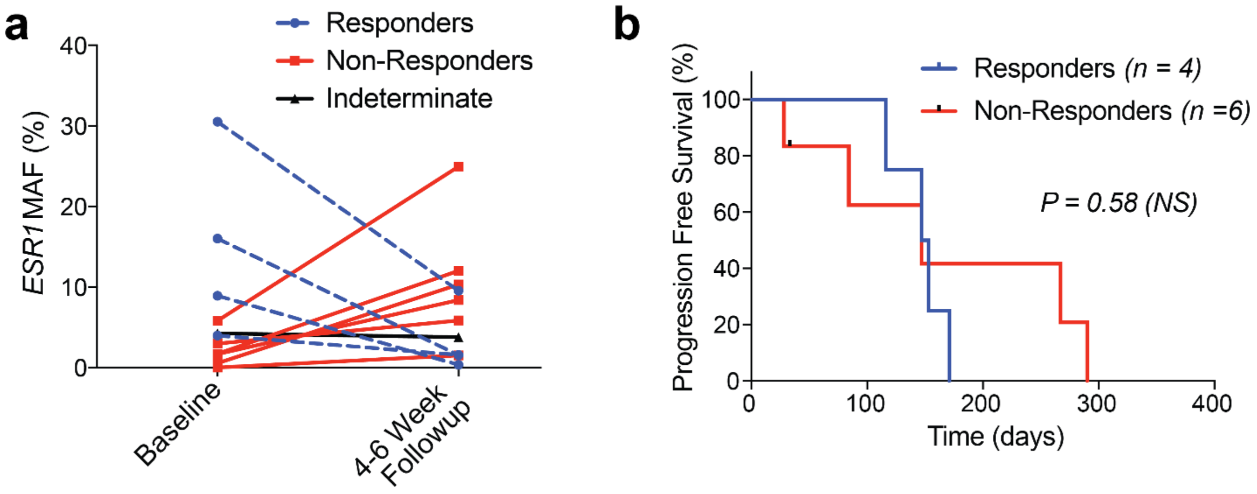

Supplementary Fig. S5: Detection of *ESR1*, *PIK3CA,* and *TP53* mutation by dPCR-SEQ. Lollipop plots showing the number (vertical axis) and position (horizontal axis) of mutations across the protein sequence of breast cancer relevant genes (*ESR1*, *PIK3CA* and *TP53*). Plot originally generated using the cBioPortal. Green circle indicates missense mutations. dPCR-SEQ was targeted to the complete coding region of *ESR1* and *TP53* whereas only hotspot regions in *PIK3CA* were targeted.

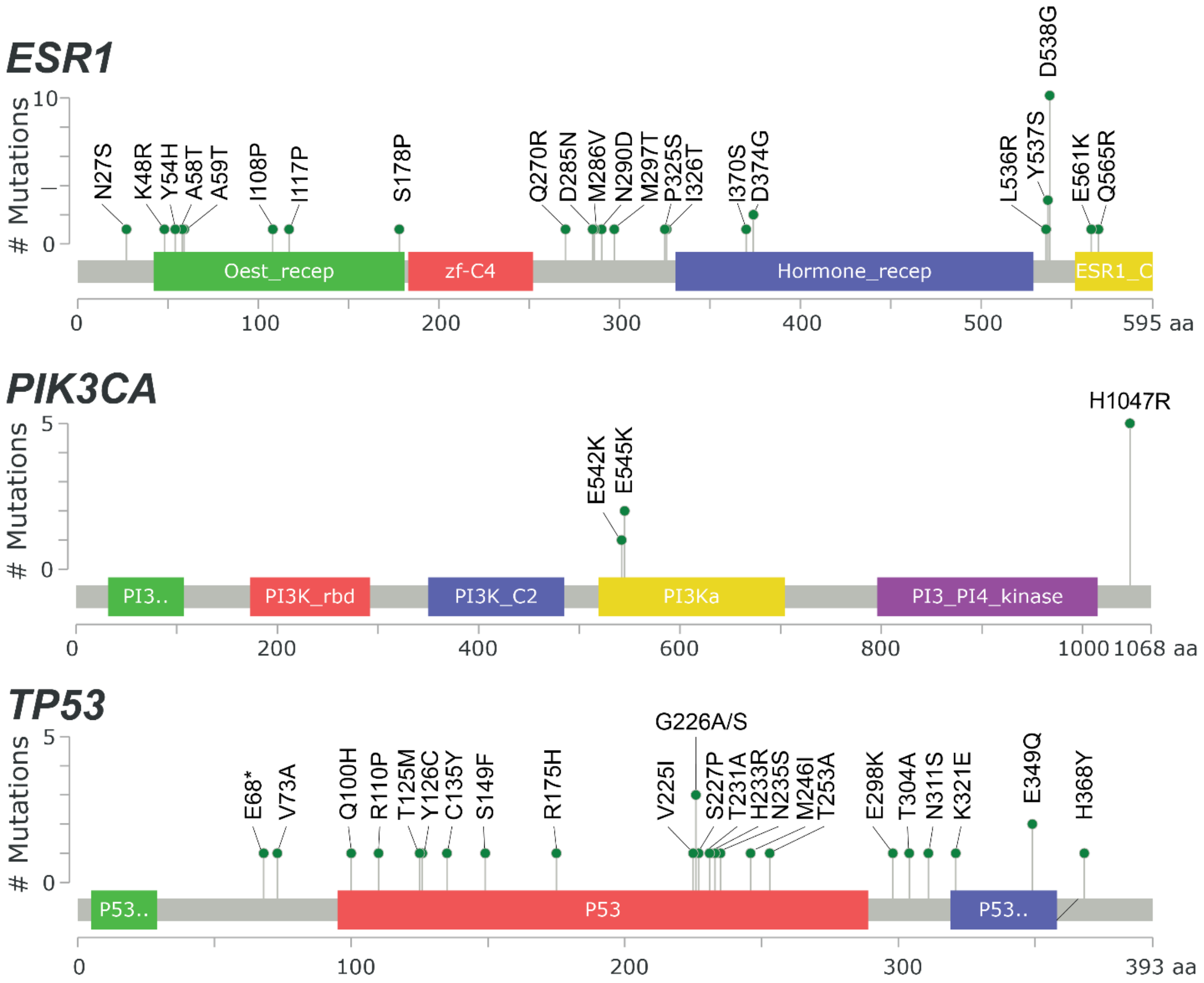

Supplement: Supplementary file 1 — Supplementary Material [file 41523_2018_93_MOESM1_ESM.pdf]
